# Anticancer Water‐Soluble Organoruthenium Complexes: Synthesis and Preclinical Evaluation

**DOI:** 10.1002/cbic.202200259

**Published:** 2022-08-03

**Authors:** Maria Azmanova, Laia Rafols, Patricia A. Cooper, Colin C. Seaton, Steven D. Shnyder, Anaïs Pitto‐Barry

**Affiliations:** ^1^ Université Paris-Saclay CNRS Institut Galien Paris-Saclay 92296 Châtenay-Malabry France; ^2^ School of Chemistry and Biosciences University of Bradford BD7 1DP Bradford UK; ^3^ Institute of Cancer Therapeutics University of Bradford BD7 1DP Bradford UK

**Keywords:** bioinorganic chemistry, half-sandwich complexes, in vivo evaluation, phosphine ligands, metallodrugs

## Abstract

The synthesis, characterisation, and evaluation of the *in vitro* cytotoxicity of five maleonitriledithiolate‐based ruthenium metal complexes bearing various phosphine ligands towards two ovarian cancer cell lines (A2780 and A2780cisR), one non‐small‐cell lung cancer cell line (H460) and one normal prostate cell line (PNT2) are presented herein. These 18‐electron complexes were designed with four water‐soluble phosphine ligands to increase the water‐solubility character of the corresponding electron‐deficient ruthenium complex which showed great *in vitro* promises, and triphenylphosphine for comparison. The complexes with triphenylphosphine‐3,3′,3′′‐trisulfonic acid and triphenylphosphine present similar cytotoxicity compared to the 16‐electron precursor, with equal cytotoxicity to both A2780 and A2780cisR. Hints at the mechanism of action suggest an apoptotic pathway based on reactive oxygen species (ROS) production. No toxicity was observed in preliminary *in vivo* pilot studies for these two complexes in subcutaneous A2780 and A2780cisR xenograft models, with some evidence of tumour growth delay.

## Graphical abstract

**Phosphine ligands** are coordinated to [(η^6^‐*p*‐cymene)Ru(maleonitriledithiolate)] to obtain ruthenium(II) complexes with different hydrophilicities. Characterisation and evaluation of the *in vitro* cytotoxicity towards two ovarian, one non‐small‐cell lung cancer cell lines and one normal prostate cell line are presented. A mechanism of action based on reactive oxygen species production leading to apoptosis is suggested. *In vivo* evaluation suggests some evidence of tumour growth delay.

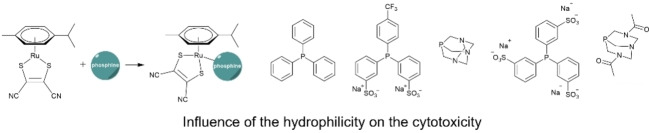

## Introduction

Cancer is the second leading cause of death worldwide, accounting for 9.6 million deaths in 2018.^[1]^ There is a need for the development and screening of anticancer therapeutics with non‐conventional mechanisms of action (MoAs).^[2]^ Half‐sandwich complexes of metals from groups eight and nine (Fe, Ru, Os; Co, Rh, Ir) are promising anticancer candidates; several complexes exhibit their anticancer properties *via* MoAs different than nuclear DNA binding while also offering an increased drug uptake and lower toxicity compared to platinum.^[3]^

Electron‐deficient half‐sandwich complexes are a class of under‐studied metallodrug candidates. Although usually unstable intermediates in catalytic processes,^[4]^ some stable coordinatively unsaturated 16‐electron (16‐e) organometallics have been reported.^[5]^ In recent years, we have developed a strong interest in elucidating the chemistry of 16‐e dithiolate‐half‐sandwich complexes of precious metals (*e. g*., thermochromism,^[6]^ reactivity,^[7]^ CO_(g)_ capture and release,^[8]^ or precursors for the fabrication of nanocrystals^[9]^).

**Figure 1:**
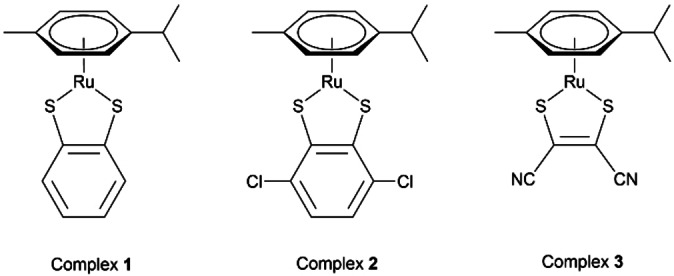
Molecular structures of complexes **1–3**.

In 2019,^[10]^ we reported the anticancer activity of such complexes against colorectal cancer *in vitro* models, determining that complexes **1** and **2** (Figure 1) exhibit significantly high cytotoxicity against colorectal cancer cell lines (12 to 34 × more potent than cisplatin). Furthermore, these two complexes exhibit high *in vitro* selectivity (>50‐fold) towards the cancer cells tested, compared to PNT2 normal cells.

In 2020,^[11]^ we reported the evaluation of the *in vitro* and *in vivo* (in mice) anticancer properties of the electron‐deficient organoruthenium complex **3** [(η^6^‐*p*‐cymene)Ru(Mnt)] (Mnt: maleonitriledithiolate) (Figure 1). This compound was found to be highly cytotoxic *in vitro*: 5 to 60 × more potent than cisplatin towards some ovarian, colon, and lung cancer cell lines. It showed no cross‐resistance and, unlike cisplatin, the remarkable *in vitro* antiproliferative activity of this compound appears to be *p53*‐independent. *In vivo* evaluation in mice with the hollow‐fibre assay across a panel of cancer cell types and in a subcutaneous H460 non‐small‐cell lung cancer xenograft model hinted at the activity of the complex.

The absence of toxicity of **1** and **3** was confirmed against non‐immortalised lymphocytes taken from the blood of healthy individuals.^[12]^ Primary human cells freshly isolated *ex‐vivo*/*in‐vitro* are a recognised surrogate model to examine responses in humans, owing to their intact metabolic system. This study also confirmed that the compounds do not induce DNA damage in healthy cells at concentrations ≫ IC_50_ values against cancer cells. This strengthens our oxidative stress‐induction hypothesis as MoA.

The discovery of such promising anticancer drug candidates (and particularly of complex **3**, which exhibited the most interesting anticancer properties) has been a crucial milestone in our efforts to develop novel antitumoral agents. However, concomitantly, the studies we carried out on such complexes led us to identify issues which impair the full translation of their *in vitro* promises into *in vivo* lead molecules: lack of aqueous‐solubility and issues in formulating the compounds for injections into mice were found to be the most limiting factors.^[11]^

Herein, we alter the chemical structure of our most promising *in vitro* lead molecule (complex **3**) to enhance its hydrophilicity and bioavailability. For doing so, we take advantage of the vacant coordination site on the 16‐electron complex **3** to add hydrophilic ligands and to synthesise the 18‐electron adducts. Owing to the high stability of the 16‐electron complex **3**, we chose phosphine‐derivatives as ligands. Indeed, we previously showed that electron‐deficient arene‐ruthenium complexes exhibit low reactivity with σ‐donor ligands but strongly coordinate with σ‐donor, π‐acceptor ligands such as phosphines to form the corresponding 18‐electron adducts.^[7]^

**Figure 2:**
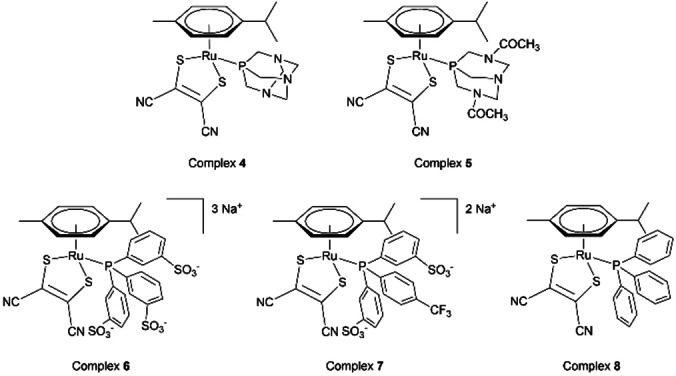
Molecular structures of complexes **4–8**.

Coordination of four water‐soluble phosphines (1,3,5‐triaza‐7‐phosphaadamantane (PTA), 3,7‐diacetyl‐1,3,7‐triaza‐5‐phosphabicyclo[3.3.1]nonane (DAPTA), triphenylphosphine‐3,3′,3′′‐trisulfonic acid trisodium salt (TPPTS), and bis(3‐sulfonatophenyl)(4‐trifluoromethylphenyl)phosphine disodium dihydrate (*p*‐DANPHOS)) are achieved to form complexes (η^6^‐*p*‐cymene)(PTA)Ru(Mnt) (**4**), (η^6^‐*p*‐cymene)(DAPTA)Ru(Mnt) (**5**), (η^6^‐*p*‐cymene)(TPPTS)Ru(Mnt) (**6**), (η^6^‐*p*‐cymene)(*p*‐DANPHOS)Ru(Mnt) (**7**), respectively (Figure 2). The highly hydrophobic triphenylphosphine is coordinated to complex **3**, leading to complex (η^6^‐*p*‐cymene)(PPh_3_)Ru(Mnt) (**8**), in order to increase further the hydrophobicity of the complex, as a mean of comparison (Figure 2).

The antiproliferative activity of complexes **4**–**8** towards ovarian (A2780 and A2780cisR) and lung (H460) cancer cells is reported, as well as towards one normal human prostate cell line (PNT2). The stability in solution is investigated, while the MoA of these metal complexes is studied *via* flow cytometry apoptotic studies, reactive oxygen species (ROS) generation, mitochondrial‐membrane potential assay, and *N*‐acetylcysteine (NAC) co‐incubation assay.

Finally, complexes **6** and **8** are progressed *in vivo* to assess toxicity and efficacy in preliminary pilot studies in mice. We chose these two complexes not only because they were the most two promising compounds identified *in vitro*, but also to test, in mice, the influence of the overall hydrophobicity of the complex: **6** being more hydrophilic than the previously studied complex **3**; **8** being more hydrophobic than **3**. The maximum tolerated doses are determined, along with the effects of **6** and **8** on human A2780 and A2780cisR subcutaneous tumour xenograft models.

## Results and Discussion

### Synthesis, characterisation and stability in solution

Complexes **4**–**8** were synthesised by stirring the 16‐electron complex **3** with an equimolar of the corresponding phosphine ligands in a solvent suitable for the ligand (dichloromethane for PTA, DAPTA, and PPh_3_; acetone for TPPTS and *p*‐DANPHOS). Complex **8** is known^[13]^ while the other complexes are new. All complexes are stable in air in their solid state and are obtained as coloured powder. Complexes **4**, **5**, and **8** are neutral, while complexes **6**–**7** are charged.

**Figure 3:**
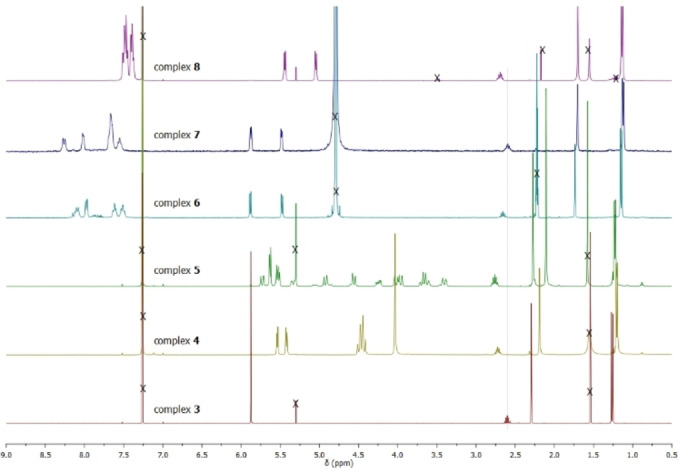
^1^H NMR spectra (400 MHz, CDCl_3_ or D_2_O) of complexes **3–8**.

^1^H, ^13^C, ^31^P{^1^H} and ^19^F{^1^H} spectroscopic data were obtained in CDCl_3_ for complexes **4**, **5**, and **8** or in D_2_O for complexes **6**–**7** depending on their solubility. In all cases, the resonances for the methyl protons (around 2.2 and 1.2 ppm) are slightly shifted upfield compared to the same protons of complex **3** while the resonance for the methine proton (around 2.6 ppm) is slightly shifted downfield (Figure 3 and Figures S1‐S16). A more noticeable change is observed for the four aromatic protons of the η^6^‐*p*‐cymene ligand which resonate as a singlet at 5.87 ppm for complex **3** and can be seen as an AB pattern shifted upfield when the phosphine ligand is added, both of which are typical of arene‐ruthenium complexes.^[14]^ ^1^H NMR analysis for complex **5** shows the non‐equivalent acyl methyl protons of the DAPTA ligand at 2.27 and 2.11 ppm. The methylene protons of the same complex exhibit a splitting pattern attributed to the diastereotopic nature of the NCH_2_N and PCH_2_N moieties.^[15]^ Again, the non‐equivalences of the quaternary carbonyl carbons (170.04 and 169.68 ppm), and acyl methyl carbons (21.73 and 21.42 ppm) are observed in the ^13^C NMR spectrum of complex **5** (Figure S5).

Coordination of the metal to the phosphine ligands happens *via* the phosphorus atom, and this is evidenced by a downfield shift of the phosphorus signal in the ^31^P{^1^H} NMR spectra of complexes **4**–**8** (Figures S3, S6, S9, S12, S16) for the spectra of the five complexes; *e. g*., from −104.3 ppm (crystalline form)^[16]^ or −96.2 ppm in MeOD solution^[17]^ for the free ligand PTA to −31.56 ppm for complex **4** in CDCl_3_; from −81.06 ppm for the free ligand DAPTA^[18]^ to −7.84 ppm for complex **5** in CDCl_3_; from −5.39 ppm in CDCl_3_ for the free ligand PPh_3_ to 34.23 ppm for complex **8** in CDCl_3_; from −8.3 ppm in D_2_O^[19]^ for the free ligand TPPTS to 38.65 ppm for complex **6** in D_2_O, as has been previously reported.^[20]^

The IR spectra of the complexes in their solid state all exhibit a C≡N stretching vibration at around 2200 cm^−1^, as is the case for [(*p*‐cymene)Ru(Mnt)] (Figure S17). Complex **5** exhibits the characteristic C=O stretching vibration at 1643 cm^−1^. High‐resolution ESI‐MS was obtained for complexes **4**–**8** and confirmed the proposed structures (Figures S18–S22).

**Figure 4:**
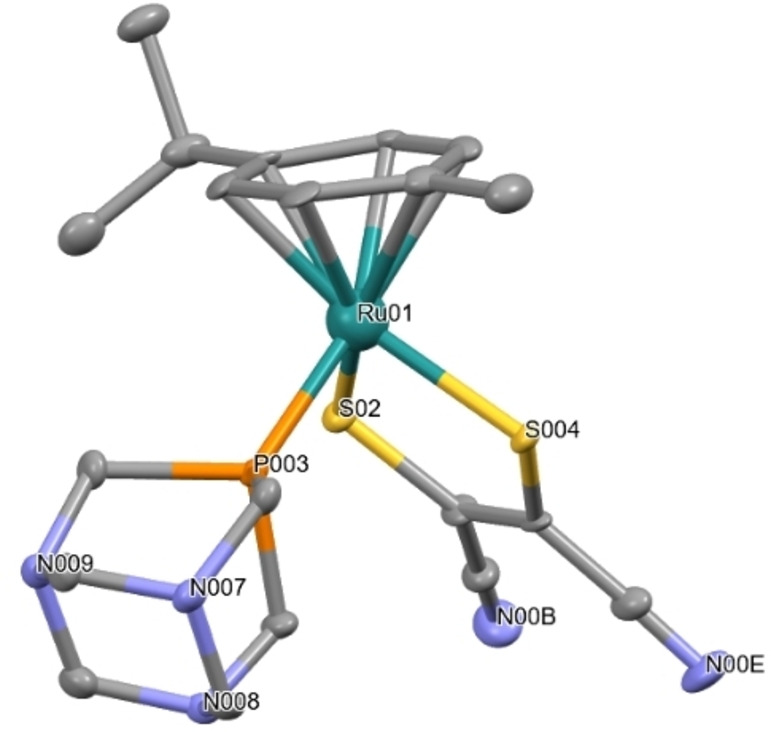
Molecular structure of complex **4**; all hydrogen atoms and the solvent molecule are omitted for clarity. Selected distances (Å) and angles (°): Ru1‐S2 2.369(2), Ru1‐S4 2.370(1), Ru1‐P3 2.279(1), S2‐Ru1‐S4 87.98(6), P3‐Ru1‐S2 87.46(6), P3‐Ru1‐S485.13(6).

The molecular structures of complexes **4** and **8** were confirmed by X‐ray single crystal diffraction. Single crystals were obtained by slow diffusion of hexane into a concentrated solution of the complex dissolved in CD_2_Cl_2_ at −18 °C. The molecular structure of complex **8** has already been reported, and both the conformation, the interatomic distances, and the bond angles were found to be similar.^[13]^ The ruthenium(II) centre in complex **4** adopts a pseudo‐octahedral structure, with Ru^II^ bound to a η^6^‐*p*‐cymene ring, a *S*,*S*‐chelated dithiolate ligand and the phosphine as ligands to form an 18‐electron complex with “piano‐stool” geometry (Figure 4). The dihedral angle RuS_2_‐S_2_C_2_ along the S⋅⋅⋅S vector is 178.95°. The metallacycle RuS_2_C_2_ is therefore not anymore planar, this is expected because of the repulsion from the PTA phosphine ligand. Selected bonds and bond angles are given in the Supporting Information (Figure S23 and Tables S1‐S3). Deposition Number 2169506 contains the supplementary crystallographic data for this paper. These data are provided free of charge by the joint Cambridge Crystallographic Data Centre and Fachinformationszentrum Karlsruhe Access Structures service www.ccdc.cam.ac.uk/structures.

The stability of the complexes in the presence of DMSO was evaluated. Depending on the water‐solubility at millimolar concentration, the complexes were dissolved in a mixture of dimethylsulfoxide and RPMI of different v/v ratio, and UV‐vis spectra were recorded at t=0 and 24 hours. The five complexes are stable under these conditions (Figures S24–S28).

### *In vitro* antiproliferative activity

Chemosensitivity studies were undertaken using a 24‐hour MTT assay, with a 72‐hour recovery period. The IC_50_ values were determined against ovarian A2780 (cisplatin‐sensitive) and A2780cisR (cisplatin‐resistant) cancer cell lines, non‐small‐cell lung H460 cancer cell line, and normal prostate PNT2 cell line, exposed to each of compounds **4**–**8**, cisplatin or the phosphine ligands (Table 1 and S4, Figures S29–S31). Complex **4** suffers from poor water‐solubility, it precipitates at concentrations higher than 50 μM in the drug‐medium solutions and IC_50_ values could not be determined. Its analogue complex **5** displays variable IC_50_ values for all tested cell lines which do not correlate together. This is unusual but can be related to solubility issues not visible for the naked eye and thus hindering its activity in cells. Complexes **6**–**8** show significantly high cytotoxicity (nanomolar and low micromolar range) for the tested cell lines indicating that the presence of the triphenylphosphine moiety in the complexes is important for the cancer‐inhibiting properties of the compounds. When tested on their own, the phosphine ligands are not active below 100 μM for all four cell lines. The lack of solubility of complex **4** did not allow for the cytotoxicity to be determined. This therefore prevents comparison with the well‐known RAPTA family, and more especially with RAPTA−C which bears a PTA ligand and a *p*‐cym ligand, making this complex relatively similar in terms of geometry with complex **4**.^[21]^ Interestingly, the 18‐electron bidentate *S,O*‐pyrithione complex with similar steric hindrance [(η^6^‐*p*‐cymene)Ru(pyrithionato)Cl] exhibits a EC_50_ value of 3.81±0.06 μM against MCF‐7 cell line,^[22]^ which is of the same order of magnitude than the 18‐electron complexes **5**–**8**, but much better than the corresponding *O,O*‐ ligand. This confirms the interest of the sulfur coordinating atom in terms of cytotoxicity for organoruthenium complexes.^[23]^

**Table 1:** IC_50_ values (μM) of complexes **3–8** against cancerous ovarian cancer (A2780), cisplatin‐resistant ovarian cancer (A2780cisR), non‐small‐cell lung cancer (H460) and non‐cancerous human prostate (PNT2) cells.

Compounds	IC_50_ values [μM]±SD			
	A2780	A2780cisR	H460	PNT2
Cisplatin	6.5 ±0.4	18.0 ±0.6	4.5 ±0.2	12.0 ±0.7
**3** ^[11]^	0.5 ±0.04	0.32 ±0.15	0.8 ±0.1	–
**4** ^[a]^	≥ 50	≥ 50	≥ 50	≥ 50
**5**	14 ±2	72 ±6	≥ 100	33 ±1.4
**6**	0.60 ±0.07	1.30 ±0.01	2.0 ±0.2	1.6 ±0.3
**7**	2.8 ±0.8	2.3 ±0.7	2.3 ±0.3	2.2 ±0.1
**8**	0.96 ±0.04	0.98 ±0.06	5.7 ±0.8	2.4 ±0.2

[a] Complex is precipitating at concentrations higher than 50 μM and IC_50_ values cannot be determined.

**Figure 5:**
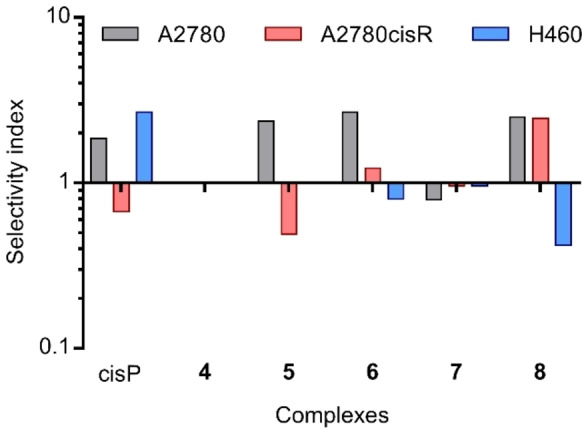
Selectivity indices (SI) of cisplatin and complexes **4–8** against A2780, A2780cisR, and H460 cancer cells.

A major limitation of clinically used anticancer drugs is their poor selectivity towards cancer cells, which creates harmful side effects for patients and therefore restricts drug dosage.^[24]^ Cell viability was determined for complexes **4**–**8** against human prostate cell line, PNT2, which is used to indicate their cancer selectivity (Table 1). Results are expressed as a selectivity index (SI), defined as the ratio of the mean IC_50_ value for normal PNT2 cells divided by the mean IC_50_ value for each individual cancer cell line (Table S5 and Figure 5). All complexes which are cytotoxic exhibit moderate SI values.

The degree of cross‐resistance was then studied. The resistance factors (RF) are the ratios of the IC_50_ values determined in the treated cell line A2780cisR and the IC_50_ values in the A2780 cell line. It is interesting to note that ciplatin exhibits some cross‐resistance, with complexes **5** and **6** having a similar selectivity factor while complexes **7** and **8** are completely non cross‐resistant (Table S6). These results suggest that the complexes have a different mode of action from that of cisplatin; such a tendency has been previously observed for arene ruthenium metal‐based drugs.^[25]^

The two most active complexes **6** and **8** have been further assessed *in vitro* using ROS detection, co‐incubation with NAC, mitochondrial membrane potential studies, and apoptosis.

### ROS generation

**Figure 6:**
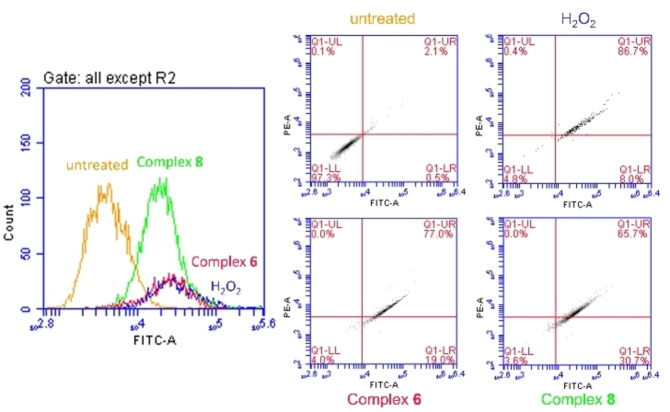
Diagram showing the four quadrants in the cell population plots; Q1‐UL=cells with elevated O2⋅^−^; Q1‐UR=cells with elevated ROS+O2⋅^−^; Q1‐LR=cells with elevated ROS; Q1‐LL=healthy cells. Populations of A2780 cells after 5 hours incubation at 37 °C with no drug added (negative control), hydrogen peroxide (positive control), and complexes **6** and **8** at their respective IC_50_ concentration.

To confirm the hypothesis of induction of oxidative stress by complexes **6** and **8**, based on the potential mechanism of action observed for complex **3**, the intracellular production of ROS in A2780 cells exposed to the complexes was investigated using the fluorescent DCFH_2_‐DA assay with the analysis performed by flow cytometry. A2780 cells were either exposed to complex **6** or **8** (IC_50_ concentrations), or to H_2_O_2_ (200 μM) as a positive control. After five hours of drug exposure, we observed a significant increase of ROS levels in treated cells compared to untreated cells (Figure 6). The activity seen for the complexes is comparable to that observed for H_2_O_2_. These observations are consistent with the proposed MoA for these complexes, which is based on the disruption of the cellular redox balance.

### Cell viability with *N*‐acetylcysteine

**Figure 7:**
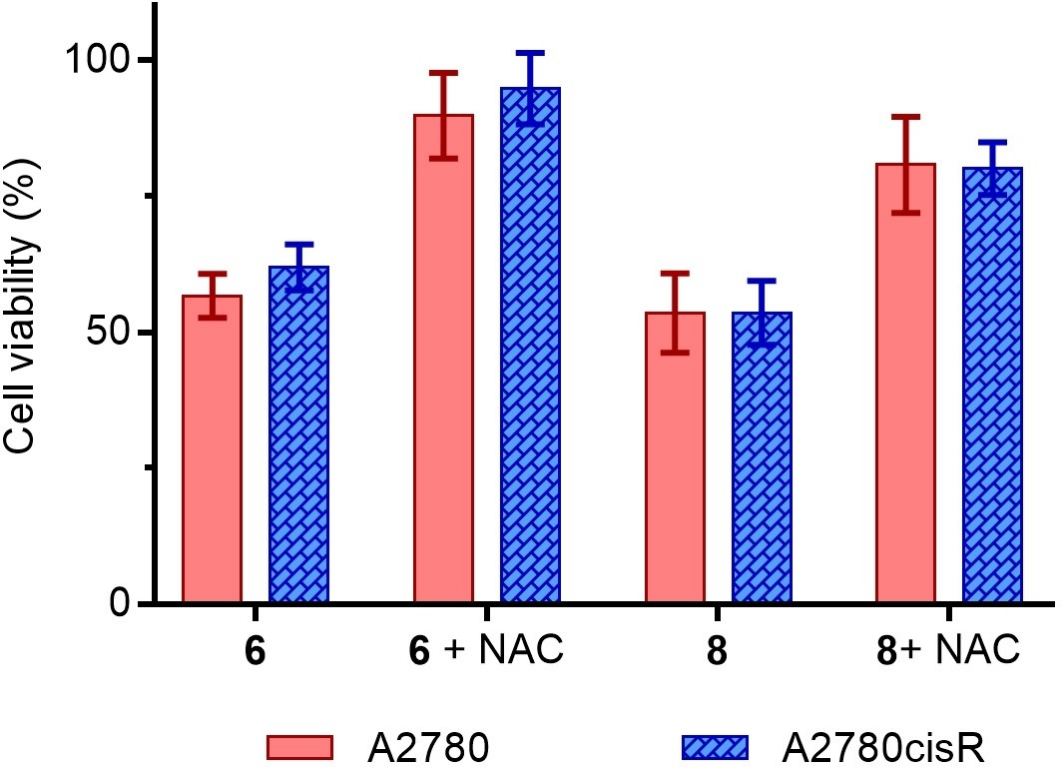
Cell viability of A2780 and A2780cisR cells at the IC_50_ concentration of complexes **6** and **8** in the presence of a large excess of NAC (5 mM).

We previously reported that the cytotoxic activity of the electron‐deficient complex **3** is inhibited by the ROS scavenger NAC, thus indicating ROS production to be part of the possible MoA for this complex.^[11]^ To test if NAC would have the same effect on the anticancer activity of complexes **6** and **8**, A2780 and A2780cisR cells were co‐incubated with a large excess of NAC (5 mM) for 30 minutes before the treatment of cells with the complexes at IC_50_ concentrations. Pre‐treatment of cells with NAC at high concentrations leads to an increase of the cell viability, which indicates that NAC is inhibiting the cytotoxic activity of complexes **6** and **8** for both cell lines, protecting the cells from the antiproliferative effect of these complexes (Figure 7). In fact, complex **6** and NAC do not react together as shown by NMR studies over the period of 24 hours (Figure S32) which therefore rules out the possibility of NAC deactivating the complexes, and strengthens our hypothesis of induction of high levels of ROS being part of the possible MoA for these complexes.

### Mitochondrial‐membrane potential assay

**Figure 8:**
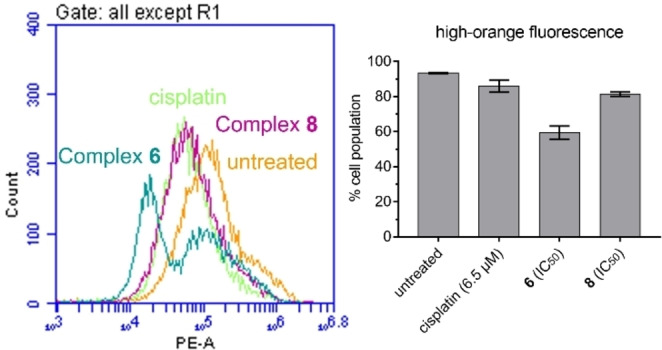
Effect of complexes **6** and **8** on the mitochondrial membrane potential. Cells were treated with complexes at IC_50_ concentrations as well as cisplatin at 6.5 μM (positive control) or untreated (negative control). The percentages of cells with depolarised mitochondrial membrane potential were assessed by flow cytometry after staining with JC‐10. The data are expressed as the means±SD of the three individual experiments.

A membrane potential sensitive fluorescent probe JC‐10 was used to determine whether the apoptosis induced by complexes **6** and **8** is accompanied by changes in the mitochondrial membrane potential (ΔΨm) of A2780 cells. JC‐10 aggregates inside mitochondria and emits orange fluorescence. Following membrane polarisation, JC‐10 is disaggregated and this in turn reduces the orange fluorescence. Treatment of A2780 cells with complexes **6** and **8** (IC_50_ concentrations), or cisplatin (6.5 μM) led to shifts of the orange fluorescence to lower intensities indicating mitochondrial depolarisation (Figure 8 and Tables S8‐S11). The populations of the ΔΨm depleted cells were comparable for complex **8** and cisplatin, and complex **6** induced higher increases of A2780 cell populations with depolarised mitochondria.

### Induction of apoptosis

**Figure 9:**
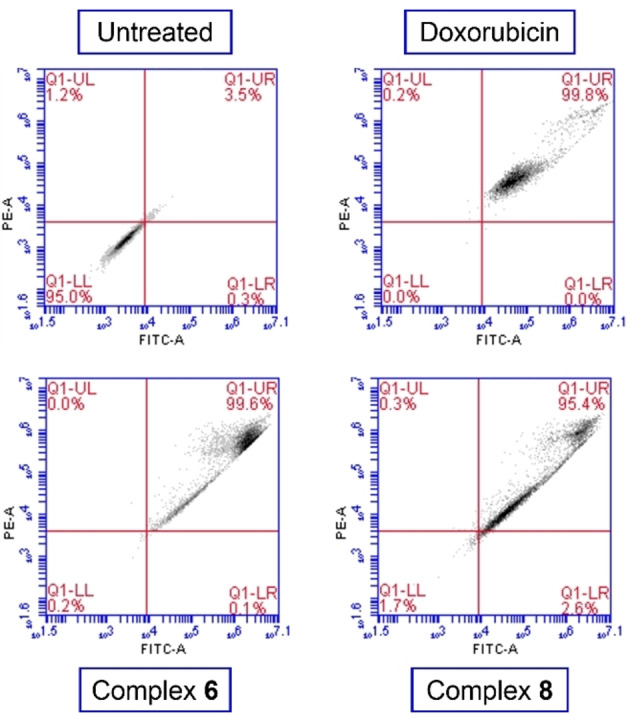
Flow cytometry analysis of A2780 cells exposed to complexes **6** or **8** at twice the IC_50_ concentration compared to a negative control. FITC reads annexin fluorescence and PE reads propidium iodide. Abbreviations: Q1‐UL=non‐viable cells; Q1‐UR=late‐stage apoptotic cells; Q1‐LR=early‐stage apoptotic cells and Q1‐LL=healthy cells.

To gain an insight into the possible MoA of the most active complexes **6** and **8** reported in this work, apoptosis studies and flow cytometry analysis of treated A2780 cells were carried out. Annexin V‐FITC and propidium iodide (PI) have been used to distinguish between viable cells (Q1‐LL), early apoptotic cells (Q1‐LR), late apoptotic cells (Q1‐UR), and necrotic cells (Q1‐UL). In this experiment, A2780 cells were treated with either complex **6** or **8** for 24 h at 2 × IC_50_ concentrations, or doxorubicin (0.5 μM) for comparative purposes (Figure 9 and Tables S12‐S14 to show the full numerical data and statistical analysis of results). Both complexes **6** and **8** induce apoptosis of A2780 cells and treatment with the complexes results in 99.6 % and 95.4 % of the cells in late apoptosis, respectively. The activity for the complexes is comparable to the activity seen for doxorubicin which is an anticancer drug known to have an apoptotic MoA.^[26]^

### In vivo studies

*In vivo* screening is rapidly developing in the field of bioinorganic chemistry, ranging from zebrafish^[27]^ to small rodents^[28]^ to study the biodistribution or the therapeutic activity of novel compounds. Compounds **6** and **8** were chosen for *in vivo* preclinical studies based on their *in vitro* potential. The maximum tolerated dose (MTD) was established at 50 mg/kg for compound **6** and 15 mg/kg for compound **8** when administered intraperitoneally (i. p.) daily for four days and monitored for a further two weeks (Figures S33–S35).

**Figure 10:**
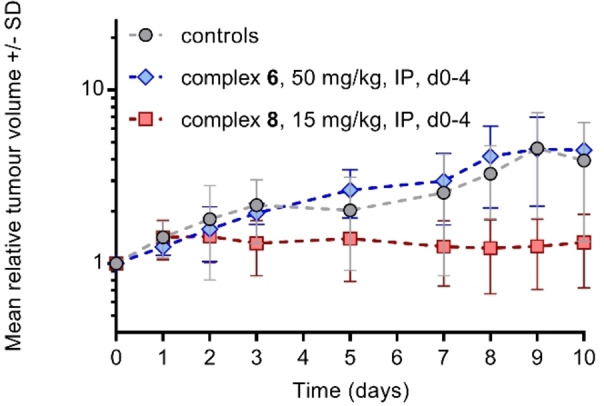
Xenograft study of the therapy of A2780cisR tumours with complexes **6** and **8** (mean relative tumour volume±SD against time).

After establishing their MTD, the efficacy of complexes **6** and **8** in A2780 and A2780cisR subcutaneous tumour xenograft models was evaluated in a preliminary pilot study. Due to the small numbers of animals used in these studies, no statistically significant growth delays were observed, however in the A2780cisR study there appeared to be a tumour growth delay in the complex **8**‐treated group (Figures 10, S36–S38 and Table S16). No growth delays were seen for complex **6** with either tumour model, or complex **8** with the A2780 model (Figure 10 and Tables S15‐S16). No toxicity was observed in either study for either compound.

## Conclusion

In conclusion, five 18‐electron phosphine‐containing half‐sandwich metal complexes based on the electron‐deficient [(η^6^‐*p*‐cymene)Ru(maleonitriledithiolate)] were synthesised and characterised. Two ionic phosphines and three neutral ones were used to obtain metal complexes of various water‐soluble character. Their stability in solution was investigated and found sufficient for *in vitro* screening, with two of the complexes being dissolved directly in the biological medium without the need of DMSO, except the (η^6^‐*p*‐cymene)(PTA)Ru(Mnt) which precipitated at concentrations higher than 50 μM. The (η^6^‐*p*‐cymene)(TPPTS)Ru(Mnt) (**6**) and (η^6^‐*p*‐cymene)(PPh_3_)Ru(Mnt) (**8**) complexes were found to be of the same activity as their 16‐electron precursor against A2780 and A2780cisR ovarian and H460 non‐small‐cell lung cancer cell lines, in the very low micromolar range. There is no cross‐resistance observed for complexes **7** and **8**. Interestingly, none of the complexes showed selectivity towards non‐small‐cell lung cancerous cells (H460) *versus* normal cells (PNT2), while a 2–3×selectivity towards cancerous ovarian cells (A2780 and A2780cisR) *versus* normal cells (PNT2) was observed for complexes **6** and **8**. The mechanism of action of the complexes appears to induce the apoptosis of cells by the intracellular production of ROS. *In vivo* results for complexes **6** and **8** with subcutaneous A2780 and A2780cisR xenograft models suggest that no toxicity was observed for any of the groups over the period of the experiment, and that there is some evidence of tumour growth delay. It is of interest to observe how the addition of a phosphine ligand can dramatically alter or not the anticancer properties of these complexes.

## Experimental Section

**Materials and instrumentations**: Roswell Park Memorial Institute (RPMI) 1640 medium, foetal bovine serum (FBS), penicillin and streptomycin, phosphate‐buffered saline (PBS, pH 7.4) and other tissue culture reagents were purchased from Gibco (Thermo Fisher Scientific, UK). Non‐dried solvents were purchased from Fischer Scientific and used as received. Dichloromethane was dried over molecular sieves. All reactions were performed under standard Schlenk conditions unless otherwise stated. All phosphines were kept under vacuum and handled under constant flow of nitrogen. [(η^6^‐*p*‐Cymene)Ru(Mnt)] was synthesised according to a previously reported method.^[13]^ All NMR spectra were recorded on a 400 MHz Bruker Spectrospin spectrometer using 5 mm NMR tubes. Deuterated solvents were purchased from Goss Scientific Instrument. The ^1^H and ^13^C NMR chemical shifts were internally referenced to TMS *via* residual solvent peaks CHCl_3_ (δ=7.26 and 77.16 ppm). Coupling constants are in Hz; abbreviations: s, singlet; d, doublet; sept, septuplet; m, multiplet. Cell lines were provided by the Institute of Cancer Therapeutics, University of Bradford, UK. Cells were incubated in a ThermoScientific HERAcell 150 incubator, and observed under a Nikon ECLIPSE TS100 Microscope.

## Synthesis

**[(η^6^**‐***p*** **‐Cymene)Ru(Mnt)PTA] (4)**: [(η^6^‐*p*‐Cymene)Ru(Mnt)] (50 mg, 0.13 mmol, 1 eq.) was placed in an oven‐dried 50 mL two‐neck round‐bottom flask equipped with a stirrer and a pressure‐equalising dropping funnel and dissolved in dry dichloromethane (15 mL) under nitrogen. In a second 50 mL two‐neck round‐bottom flask 1,3,5‐triaza‐7‐phosphaadamantane (PTA) (21 mg, 0.13 mmol, 1 eq.) was dissolved in dry dichloromethane (15 mL) under nitrogen. The PTA solution was then transferred to the dropping funnel and added slowly to the solution of [(η^6^‐*p*‐cymene)Ru(Mnt)]. The reaction was stirred at room temperature, under nitrogen, for 3 hours. The crude product was purified by column chromatography with CH_2_Cl_2_: MeOH (95 : 5 v/v) as the eluent to obtain the pure product as an orange powder (57.5 mg, 81 %). ^1^H NMR (400 MHz, CDCl_3_): *δ* 5.54 (2H, d, ^3^ *J*_H‐H_=6.1 Hz, H3), 5.42 (2H, d, ^3^ *J*_H‐H_=6.2 Hz, H4), 4.46 (6H, m, H11), 4.03 (6H, s, H10), 2.72 (1H, sept, ^3^ *J*_H‐H_=6.9 Hz, H6), 2.19 (3H, s, H1), 1.20 (6H, d, ^3^ *J*_H‐H_=6.9 Hz, H7) ppm. ^13^C NMR (100 MHz, CDCl_3_): *δ* 125.80 (C8), 116.83 (C9), 113.27 (C5), 105.36 (C2), 91.60 (C3), 91.43 (C4), 73.50 (d, C11), 52.39 (d, C10), 31.13 (C6), 22.91 (C7), 18.88 (C1) ppm. ^31^P{^1^H} NMR (161 MHz, CDCl_3_): *δ* −31.56 ppm. HRMS‐ESI^+^: calculated [M+H]^+^ 533.04106 *m/z*, found 533.04289 *m/z*.

**[(η^6^**‐***p*** **‐Cymene)Ru(Mnt)DAPTA] (5)**: [(η^6^‐*p*‐Cymene)Ru(Mnt)] (30 mg, 0.08 mmol, 1 eq.) was placed in an oven‐dried 50 mL two‐neck round‐bottom flask equipped with a stirrer and a pressure‐equalising dropping funnel and dissolved in dry dichloromethane (10 mL) under nitrogen. In a second 50 mL two‐neck round‐bottom flask 3,7‐diacetyl‐1,3,7‐triaza‐5‐phosphabicyclo[3.3.1]nonane (DAPTA) (18.3 mg, 0.08 mmol, 1 eq.) was dissolved in dry dichloromethane (10 mL) under nitrogen. The DAPTA solution was then transferred to the dropping funnel and added slowly to the solution of [(η^6^‐*p*‐cymene)Ru(Mnt)]. The reaction was stirred at room temperature, under nitrogen, for 3 hours. The crude product was purified by column chromatography with CH_2_Cl_2_:EtOH (95 : 5 v/v) as the eluent to obtain the pure product as an orange solid (40.6 mg, 84 %). ^1^H NMR (400 MHz, CDCl_3_): *δ* 5.73 (1H, d, ^3^ *J*_H‐H_=14.1 Hz, H11), 5.63 (2H, d, ^3^ *J*_H‐H_=6.3 Hz, H3), 5.53 (2H, m, H4), 5.33 (1H, d, ^3^ *J*_H‐H_=15.4 Hz, H10), 4.92 (1H, d, ^3^ *J*_H‐H_=14.0 Hz, H11), 4.56 (1H, d, ^3^ *J*_H‐H_=14.0 Hz, H10), 4.25 (1H, m, H11), 4.01 (1H, d, ^3^ *J*_H‐H_=15.1 Hz, H10), 3.96 (1H, d, ^3^ *J*_H‐H_=14.1 Hz, H11), 3.66 (2H, m, H10), 3.40 (1H, d, ^3^ *J*_H‐H_=15.3, H10), 2.76 (1H, sept, ^3^ *J*_H‐H_=6.88 Hz, H6), 2.27 (3H, s, H1), 2.11 (6H, 2 s, H13), 1.23 (6H, dd, ^3^ *J*_H‐H_=6.9, 1.7 Hz, H7) ppm. ^13^C NMR (100 MHz, CDCl_3_): *δ* 170.04 (C12), 169.68 (C12), 126.42 (C8), 125.87 (C8), 116.54 (C9), 114.96 (C9), 114.92 (C5), 106.56 (C2), 92.96 (C3), 92.90 (C3), 91.89 (C3), 91.95 (C3), 91.79 (C4), 91.74 (C4), 91.58 (C4), 91.54 (C4), 67.47 (C11), 62.32 (C11), 45.64 (C10), 45.31 (C10), 44.45 (C11), 44.23 (C10), 41.31 (C10), 41.10 (C10), 31.10 (C6), 22.90 (C7), 22.75 (C7), 21.73 (C13), 21.42 (C13), 19.07 (C1) ppm. ^31^P{^1^H} NMR (161 MHz, CDCl_3_): *δ* −7.84 ppm. HRMS‐ESI^+^: calculated [M+H]^+^ 605.0622 *m/z*, found 605.0623 *m/z*.

**[(η^6^**‐***p*** **‐Cymene)Ru(Mnt)TPPTS] (6)**: [(η^6^‐*p*‐Cymene)Ru(Mnt)] (80 mg, 0.21 mmol, 1 eq.) was placed in an oven‐dried 50 mL two‐neck round‐bottom flask equipped with a stirrer and a pressure‐equalising dropping funnel and dissolved in degassed acetone (20 mL) under nitrogen. In a second 50 mL two‐neck round‐bottom flask triphenylphosphine‐3,3’,3’’‐trisulfonic acid trisodium salt (TPPTS) (121 mg, 0.21 mmol, 1 eq.) was dissolved in degassed acetone (20 mL) and a few drops of degassed water under nitrogen. The TPPTS solution was then transferred to the dropping funnel and added slowly to the solution of [(η^6^‐*p*‐cymene)Ru(Mnt)]. The reaction was stirred at room temperature, under nitrogen, for 4 hours. The crude product was dissolved in deuterated water and filtered through celite. The resulting product was dissolved in deuterated water and purified by gel chromatography using PD MidiTrap Sephadex G‐10 columns to obtain the pure product as a dark red glossy solid (58 mg, 29 %). ^1^H NMR (400 MHz, D_2_O): *δ* 8.08 (3H, d, ^3^ *J*_H‐H_=9.4 Hz, H_arom_), 7.95 (3H, d, ^3^ *J*_H‐H_=7.0 Hz, H_arom_), 7.62 (3H, m, H_arom_), 7.51 (3H, m, H_arom_), 5.87 (2H, d, ^3^ *J*_H‐H_=6.2 Hz, H3), 5.46 (2H, d, ^3^ *J*_H‐H_=6.0 Hz, H4), 2.62 (1H, sept, ^3^ *J*_H‐H_=6.7 Hz, H6), 1.71 (3H, s, H1), 1.12 (6H, d, ^3^ *J*_H‐H_=6.8 Hz, H7) ppm. ^13^C NMR (100 MHz, D_2_O): *δ* 143.26 (C12), 138.46 (C10), 136.69 (CH_arom_), 132.00 (CH_arom_), 128.90 (CH_arom_), 128.83 (CH_arom_), 128.09 (CH_arom_), 126.39 (C8), 117.34 (C5 and C9), 95.83 (C3), 91.20 (C4), 29.58 (C6), 21.22 (C7), 17.46 (C1) ppm. ^31^P{^1^H} NMR (161 MHz, D_2_O): *δ* 38.65 ppm. HRMS‐ESI^+^: calculated [M‐3Na+3H+NH_4_]^+^ 895.96017 *m/z*, found 895.95820 *m/z*.

**[(η^6^**‐***p*** **‐Cymene)Ru(Mnt)p‐DANPHOS] (7)**: [(η^6^‐*p*‐Cymene)Ru(Mnt)] (40 mg, 0.11 mmol, 1 eq.) was placed in an oven‐dried 50 mL two‐neck round‐bottom flask equipped with a stirrer and a pressure‐equalising dropping funnel and dissolved in degassed acetone (15 mL) under nitrogen. In a second 50 mL two‐neck round‐bottom flask bis(3‐sulfonatophenyl)(4‐trifluoromethylphenyl)phosphine disodium dihydrate (*p*‐DANPHOS) (57 mg, 0.11 mmol, 1 eq.) was dissolved in degassed acetone (15 mL) and a few drops of degassed water under nitrogen. The *p*‐DANPHOS solution was then transferred to the dropping funnel and added slowly to the solution of [(η^6^‐*p*‐cymene)Ru(Mnt)]. The reaction was stirred at room temperature, under nitrogen, for 4 hours. The crude product was dissolved in deuterated water and filtered through celite. The resulting product was dissolved in deuterated water and purified by gel chromatography using PD MidiTrap Sephadex G‐10 columns to obtain the pure product as a dark red glossy solid (35 mg, 36 %). ^1^H NMR (400 MHz, D_2_O): *δ* 8.26 (2H, d, ^3^ *J*_H‐H_=10.6 Hz, H15), 8.01 (2H, d, ^3^ *J*_H‐H_=5.0 Hz, H11), 7.66 (6H, m, H13, H14, and H18), 7.55 (2H, t, ^3^ *J*_H‐H_=8.12 Hz, H17), 5.87 (2H, d, ^3^ *J*_H‐H_=5.6 Hz, H3), 5.48 (2H, d, ^3^ *J*_H‐H_=5.6 Hz, H4), 2.59 (1H, sept, ^3^ *J*_H‐H_=6.6 Hz, H6), 1.70 (3H, s, H1), 1.12 (6H, d, ^3^ *J*_H‐H_=6.8 Hz, H7) ppm. ^13^C NMR (100 MHz, D_2_O): *δ* 137.34 (CH_arom_), 134.97 (C17), 133.09 (C15), 129.84 (CH_arom_), 128.79 (C11), 126.90 (C8), 123.92 (CH_arom_), 117.61 (C5 and C9), 109.61 (CF_3_), 95.90 (C3), 91.50 (C4), 30.15 (C6), 21.59 (C7), 17.76 (C1) ppm. ^31^P{^1^H} NMR (161 MHz, D_2_O): *δ* 37.27 ppm. ^19^F{^1^H} NMR (376 MHz, D_2_O): *δ* −63.01 ppm. HRMS‐ESI^+^: calculated [M+H]^+^ 910.92807 *m/z*, found 910.92698 *m/z*.

**[(η^6^**‐***p*** **‐Cymene)Ru(Mnt)PPh_3_] (8)**: [(η^6^‐*p*‐Cymene)Ru(Mnt)] (30 mg, 0.08 mmol, 1 eq.) was placed in an oven‐dried 50 mL two‐neck round‐bottom flask equipped with a stirrer and a pressure‐equalising dropping funnel and dissolved in dry dichloromethane (10 mL) under nitrogen. In a second 50 mL two‐neck round‐bottom flask triphenylphosphine (21 mg, 0.08 mmol, 1 eq.) was dissolved in dry dichloromethane (10 mL) under nitrogen. The triphenylphosphine solution was then transferred to the dropping funnel and added slowly to the solution of [(η^6^‐*p*‐cymene)Ru(Mnt)]. The reaction was stirred at room temperature, under nitrogen, for 4 hours. The crude product was purified by column chromatography with CH_2_Cl_2_: MeOH (95 : 5) as the eluent to obtain the pure product as a dark red powder (49 mg, 96 %). ^1^H NMR (400 MHz, CDCl_3_): *δ* 7.56‐7.35 (15H, m, H11‐13), 5.44 (2H, d, ^3^ *J*_H‐H_=6.1 Hz, H3), 5.04 (2H, d, ^3^ *J*_H‐H_=6.1 Hz, H4), 2.69 (1H, sept, ^3^ *J*_H‐H_=7.0 Hz, H6), 1.70 (3H, s, H1), 1.14 (6H, d, ^3^ *J*_H‐H_=6.9 Hz, H7) ppm. ^13^C NMR (100 MHz, CDCl_3_): *δ* 134.89 (CH_arom_), 134.81 (CH_arom_), 132.09 (C10), 131.61 (C10), 130.79 (CH_arom_), 127.97 (CH_arom_), 127.88 (CH_arom_), 125.98 (C8), 116.81 (C9 and C5), 106.68 (C2), 94.49 (C3), 94.44 (C3), 90.55 (C4), 29.92 (C6), 22.21 (C7), 18.41 (C1) ppm. ^31^P{^1^H} NMR (161 MHz, CDCl_3_): *δ* 34.23 ppm. HRMS‐ESI^+^: calculated [M+H]^+^ 638.05532 *m/z*, found 638.05772 *m/z*.

**Chemosensitivity assay**: *In vitro* chemosensitivity tests were performed against A2780, A2780cisR, PNT2 and H460 cells. Cells were routinely maintained as monolayer cultures in RPMI 1640 medium supplemented with 10 % foetal calf serum, penicillin (100 I.U./mL) and streptomycin (100 μg/mL), sodium pyruvate (1 mM) and L‐glutamine (2 mM). For chemosensitivity studies, cells were seeded in 96‐well plates at a concentration of 7.5×10^3^ cells per well and the plates were incubated for 24 h at 37 °C and a 5 % CO_2_ humidified atmosphere prior to drug exposure.

Complexes were dissolved in either DMSO (complexes **4**, **5**, and **8**) or PBS (complexes **6** and **7**) to provide stock solutions which were further diluted with media to provide a range of final concentrations. Drug‐media solutions were added to cells (the final concentration of DMSO was less than 1 % (*v/v*) in all cases) and incubated for 24 h at 37 °C and a 5 % CO_2_ humidified atmosphere. The drug‐media solution was removed from the wells and the cells were washed with PBS (100 μL, twice) and 200 μL of complete fresh media were added to each well. The plates were further incubated for 72 h at 37 °C and a 5 % CO_2_ humidified atmosphere to allow for a period of recovery. 3‐(4,5‐dimethylthiazol‐2‐yl)‐2,5‐diphenyltetrazolium bromide (MTT) (40 μL, 2.5 mg/mL) was added to each well and plates were incubated for 2 h. All solutions were then removed and 100 μL of DMSO was added to each well in order to dissolve the purple formazan crystals. A Thermo Scientific Multiskan EX microplate photometer was used to measure the absorbance in each well at 570 nm. Cell survival was determined as the absorbance of treated cells divided by the absorbance of controls and expressed as a percentage. The IC_50_ values were determined from plots of % survival against drug concentration. Each experiment was repeated in triplicates of triplicates and a mean value was obtained and stated as IC_50_ (μM) ± SD. Untreated cells were used as a negative control, and cells treated with cisplatin were used as a positive control.

**Cell viability with** ***N*** **‐acetylcysteine**: Cells were seeded in 96‐well plates at a concentration of 7.5×10^3^ cells per well and the plates were incubated for 24 h at 37 °C and a 5 % CO_2_ humidified atmosphere prior to drug exposure. Complexes were dissolved in either DMSO or PBS to provide stock solutions which were further diluted with media to provide a range of final concentrations. Solution of *N*‐acetylcysteine (NAC) in media was also prepared. Cells were first treated with NAC (5 mM), incubated for 30 min, and then treated with the complexes at IC_50_ concentrations. Cells were incubated for 24 h at 37 °C and a 5 % CO_2_ humidified atmosphere. 3‐(4,5‐dimethylthiazol‐2‐yl)‐2,5‐diphenyltetrazolium bromide (MTT) (20 μL, 2.5 mg/mL) was added to each well and plates were incubated for 2 h. All solutions were then removed and 100 μL of DMSO was added to each well in order to dissolve the purple formazan crystals. A Thermo Scientific Multiskan EX microplate photometer was used to measure the absorbance in each well at 570 nm. Cell survival was determined as the absorbance of treated cells divided by the absorbance of controls and expressed as a percentage.

**FITC annexin‐V apoptosis assay**: Flow cytometry analysis of apoptotic populations was carried out using the FITC Annexin V Apoptosis Detection Kit with PI (BioLegend®) according to manufacturer's instructions. Briefly, A2780 cells were seeded in 6‐well plates at a concentration of 1.0×10^6^ cells per well and the plates were incubated for 24 h at 37 °C and a 5 % CO_2_ humidified atmosphere. Cells were washed with PBS (1 mL), treated with complexes at 2 × IC_50_ concentrations or doxorubicin (0.5 μM) as a positive control and incubated for 24 h. Cells were then harvested using trypsin (without EDTA) and stained with PI/Annexin V‐FITC. After staining in the dark for 15 min at room temperature, cell pellets were analysed in a BD Accuri C6 Plus System flow cytometer and data was analysed using the BD Accuri C6 Plus software. These experiments were carried out in duplicate of triplicates in independent experiments; although only selected dot plots are shown, full numerical data and statistical analysis can be found in the Supporting Information.

**ROS detection**: Flow cytometry analysis of total induction of ROS in A2780 cells caused by exposure to complexes **6** and **8** was carried out using DCFH_2_‐DA assay. Briefly, 1.0×10^6^ cells per well were seeded in a 6‐well plate and incubated for 24 at 37 °C and a 5 % CO_2_ humidified atmosphere. After removal of the media, cells were washed with PBS and loaded with DCFH_2_‐DA dye (20 μM, in basal RPMI). After 1 h of incubation, supernatants were removed by suction and cells were washed with PBS twice before treatment with either H_2_O_2_ (100 μM) and complexes **6** and **8** at IC_50_ concentrations (all in basal RPMI) for a period of 5 h. Cells were washed with PBS and harvested with trypsin. Cells were analysed in a BD Accuri C6 Plus System Flow Cytometer using Ex/Em: 490/525 nm for the oxidative stress detection. Data was analysed using the BD Accuri C6 Plus software. This experiment was carried out in three independent repetitions; although only selected dot plots are shown, full numerical data and statistical analysis can be found in the Supporting Information.

**Mitochondrial membrane potential assay**: Analysis of the changes of mitochondrial potential in A2780 cells after exposure to complexes **6** and **8** was carried out using the JC‐10 mitochondrial membrane potential assay kit (abcam®) according to the manufacturer's instructions. Briefly, 1.0×10^6^ cells per well were seeded in a 6‐well plate and incubated for 24 at 37 °C and a 5 % CO_2_ humidified atmosphere. Cells were washed with PBS (1 mL) once and treated with either cisplatin (6.5 μM) or complexes **6** and **8** at IC_50_ concentrations and incubated for 24 h. Supernatants were removed by suction, and each well was washed with PBS before detaching the cells using trypsin. Staining of the samples was done in tubes protected from light, incubating for 30 min in the dark at room temperature. Samples were immediately analysed on a BD Accuri C6 Plus System Flow Cytometer, reading the reduction of fluorescence in the FL2 channel.

## In vivo studies

**Compounds**: All compounds were provided in powdered form and stored in the −20 °C freezer. They were formulated just before use. Complex **8** was dissolved firstly in dimethyl sulfoxide (DMSO) in a volume equal to 20 % of the final concentration, vortexed, and diluted in arachis oil to produce the required dose. Complex **6** was dissolved in sterile water to produce the required dose. The drugs were then administered within 10 minutes of initial dilution.

**Animals**: Female Balb/c immunodeficient nude mice aged 6–12 weeks were used (Envigo, Blackthorn, UK). Mice were kept in cages housed in isolation cabinets in an air‐conditioned room with regular alternating cycles of light and darkness. They received Teklad 2018 diet (Envigo, Blackthorn, UK) and water ad libitum. All animal procedures were carried out under a project licence issued by the UK Home Office and UK National Cancer Research Institute Guidelines for the Welfare of Animals were followed throughout.

**Evaluation of MTD**: The compounds were prepared fresh on the days of treatment as described above and administered i. p. to groups of two mice in a volume of 0.1 mL per 10 g body weight. The first day of treatment was denoted as day 0. Compounds were administered to non‐tumour‐bearing animals on days 0, 1, 2, 3 and 4. A group of two untreated control animals was included in each MTD study.

Following treatment, body weight was measured on a regular basis, and behaviour and general appearance monitored visually to assess for deleterious effects (*e. g*., dehydration, impaired mobility, hunched posture, low body temperature, ulceration and significant body weight loss), with any effects during the study recorded. If body weight loss was >15 % over a 72‐hour period or if animal behaviour and appearance were significantly altered, then mice were immediately sacrificed by cervical dislocation. If no deleterious effects were seen after at least 16 days of study, then the animals were sacrificed and the dose considered non‐toxic.

**S.c. xenograft tumours**: A2780/WT and A2780/cis tumours were excised from a donor animal, placed in sterile physiological saline containing antibiotics and cut into small fragments of approximately 5 mm^3^. Under brief general inhalation anaesthesia, fragments were implanted in the flank of each mouse using a trocar. Once the tumours could accurately be measured by callipers, the mice were allocated into groups (control or treated) by restricted randomisation to keep group mean tumour size variation to a minimum, with 3 animals set up per group.

**Evaluation of efficacy in s.c. xenograft models**: The compounds were prepared fresh on the days of treatment as described above and administered i. p. to mice on days 0–4 in a volume of 0.1 mL per 10 g of body weight. Complex **8** was administered at 15 mg/kg/dose and complex **6** at 50 mg/kg/dose. The negative control group was untreated.

The effects of therapy were assessed by frequent monitoring growth of the tumours and body weight. Two‐dimensional calliper measurements of the tumours were taken, and volumes calculated using the formula (a^2^×b)/2, where a is the smaller and b the larger diameter of the tumour. Tumour volume was then normalised to the respective volume on day 0, and semi‐log plots of relative tumour volume (RTV) *versus* time were made. Mann‐Whitney U tests were performed to determine the statistical significance of any differences in growth rate (based on tumour volume doubling time, RTV2) between control and treated groups.

## Conflict of interest

The authors declare no conflict of interest.

1

## Biographical Information

*Anaïs Pitto‐Barry obtained her PhD degree from the Université de Neuchâtel (Switzerland) with Prof. R. Deschenaux. In 2012, she joined the University of Warwick (UK) to work with Profs R. K. O′Reilly FRS and A. P. Dove with a Swiss National Science Foundation Fellowship. She then worked with Profs P. J. Sadler FRS and N. P. E. Barry on the applications of metallated compounds at the University of Warwick and the University of Bradford before taking up an assistant professorship at Bradford.. She was appointed as tenured CNRS Researcher (chargée de recherche) at Institut Galien Paris‐Saclay (Université Paris‐Saclay, France) in 2021. Her research activities focus on the design of polymers used as cargo to carry organometallic complexes for medical applications*.

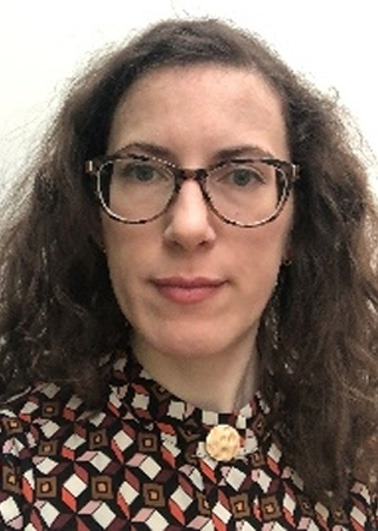

## Supporting information

As a service to our authors and readers, this journal provides supporting information supplied by the authors. Such materials are peer reviewed and may be re‐organized for online delivery, but are not copy‐edited or typeset. Technical support issues arising from supporting information (other than missing files) should be addressed to the authors.

Supporting Information

## Data Availability

The data that support the findings of this study are available in the supplementary material of this article.
