# Supplementary material for: Anticancer Water‐Soluble Organoruthenium Complexes: Synthesis and Preclinical Evaluation
Source: Chembiochem. 2022 Aug 3;23(18):e202200259. doi: 10.1002/cbic.202200259 (PMC9545474; doi:10.1002/cbic.202200259)
Supplement: Supplementary file 1 — Supporting Information [file CBIC-23-0-s001.pdf]

ChemBioChem

Supporting Information

Anticancer Water-Soluble Organoruthenium Complexes: Synthesis and Preclinical Evaluation

Maria Azmanova, Laia Rafols, Patricia A. Cooper, Colin C. Seaton, Steven D. Shnyder, and Anaïs Pitto-Barry*

Contents

I. NMR spectra for complexes **4 – 8** 2
a. NMR spectra for complex **4** 2
b. NMR spectra for complex **5** 3
c. NMR spectra for complex **6** 5
d. NMR spectra for complex **7** 6
e. NMR spectra for complex **8** 8
II. IR spectra for complexes **4 – 8** 10
III. HR-MS spectra for complexes **4 – 8** 10
IV. XRD data for complex **4** 13
V. Stability in solution for complexes **4 – 8** 15
VI. *In vitro* antiproliferative activity for complexes **4 – 8** 17
a. IC_{50} data 17
b. IC_{50} data of ligands 18
c. Selectivity index (SI) 19
d. ROS 20
e. Mitochondrial-membrane potential 21
f. Apoptosis 22
VII. *In vivo* results for complexes 6 and 8 23
a. Evaluation of the maximum tolerated dose (MTD) 23
b. Efficacy with subcutaneous tumour xenograft models 25

I. NMR spectra for complexes **4 – 8**

a. NMR spectra for complex **4**

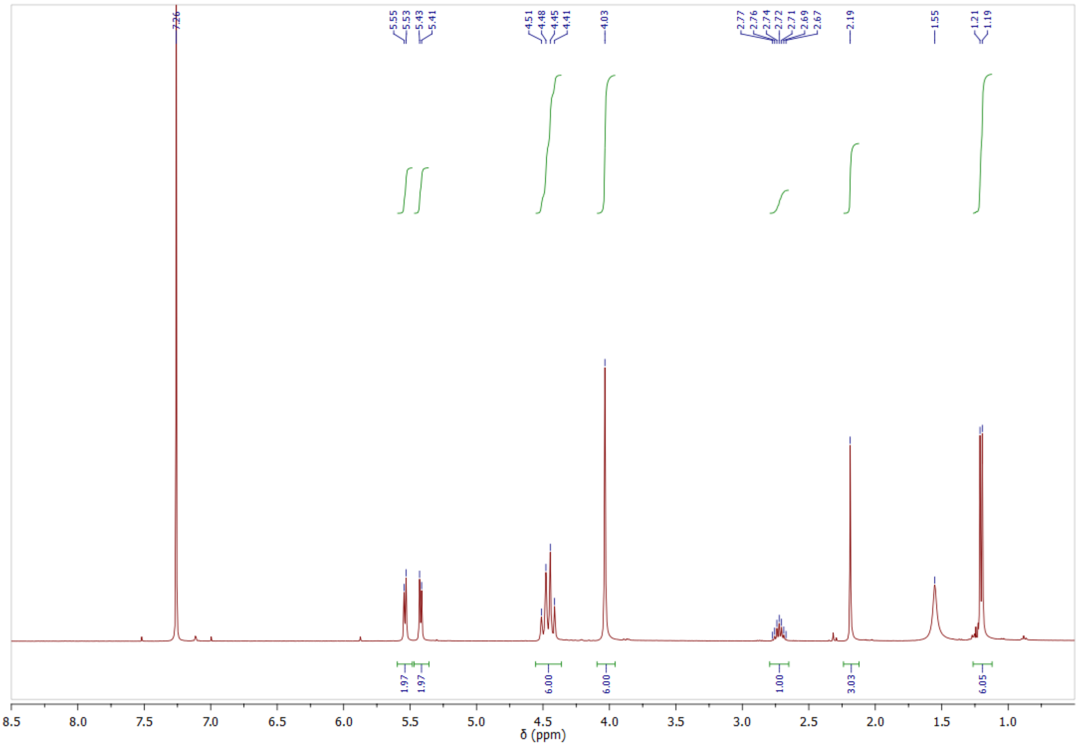

Figure S1. ^{1}H NMR spectrum (400 MHz, $CDCl_3$) of complex **4**.

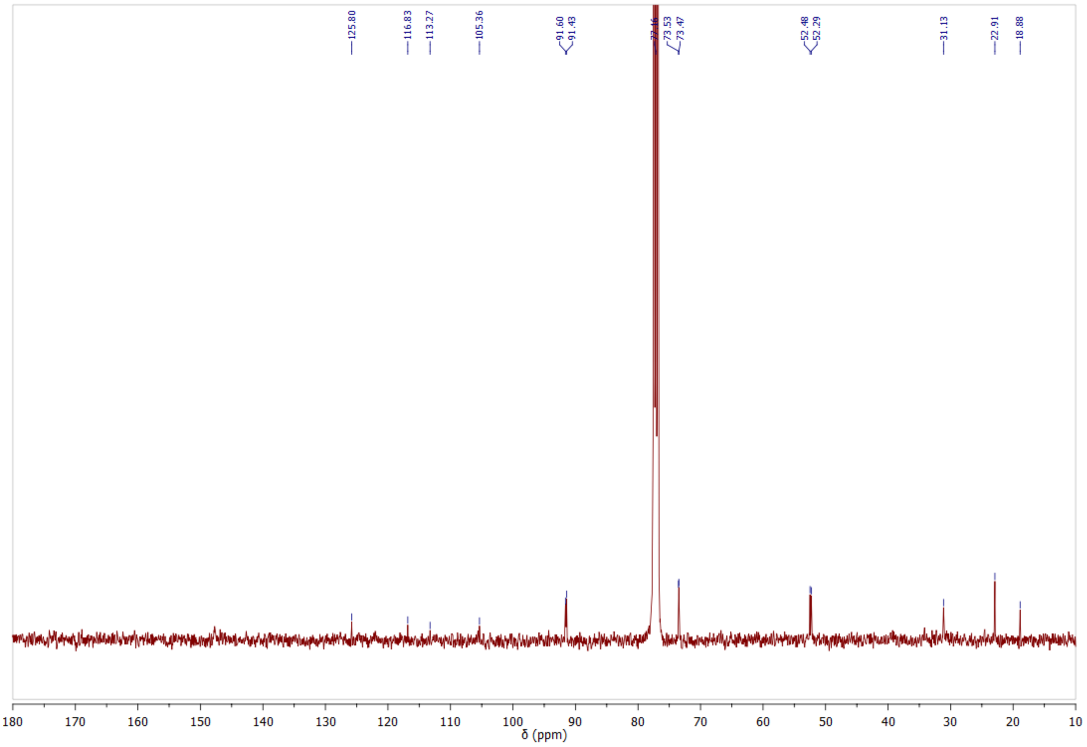

Figure S2. ^{13}C NMR spectrum (100 MHz, $CDCl_3$) of complex **4**.

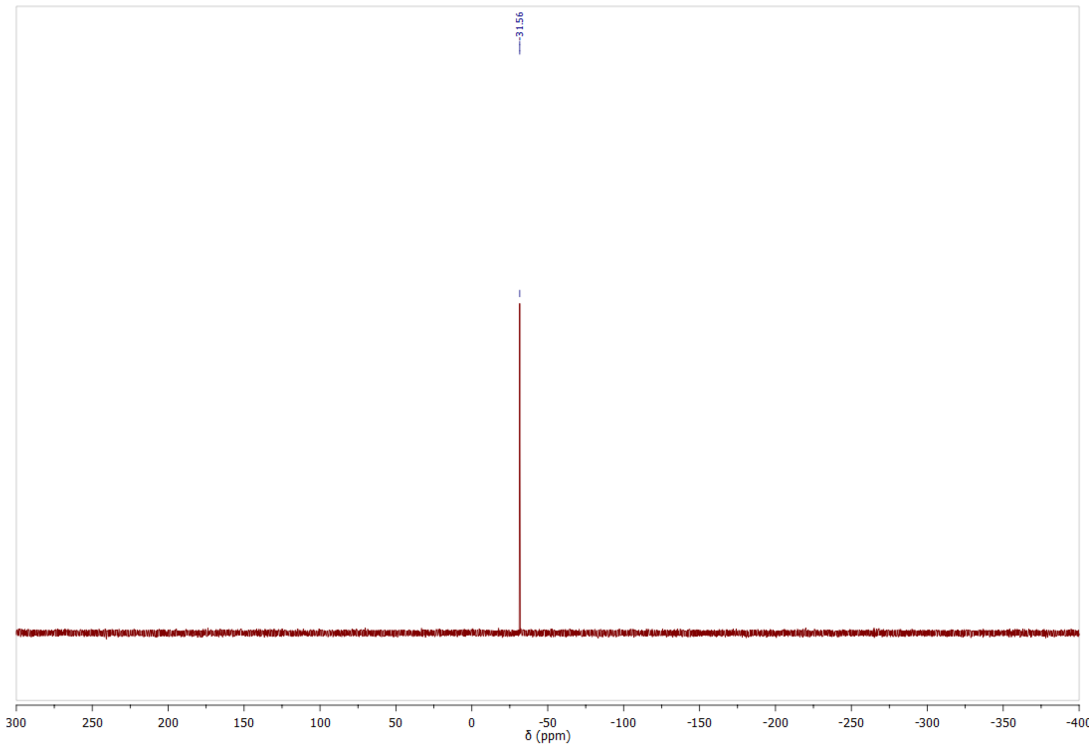

Figure S3. ^{31}P NMR spectrum (161 MHz, $CDCl_3$) of complex **4**.

b. NMR spectra for complex **5**

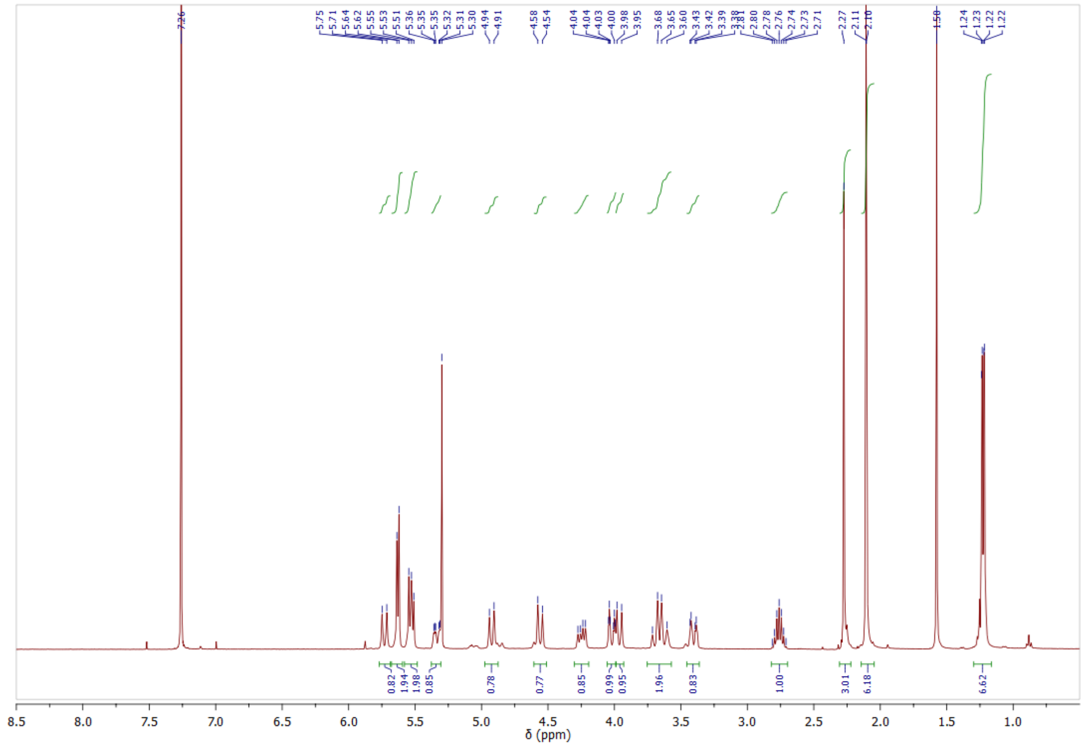

Figure S4. ^{1}H NMR spectrum (400 MHz, $CDCl_3$) of complex **5**.

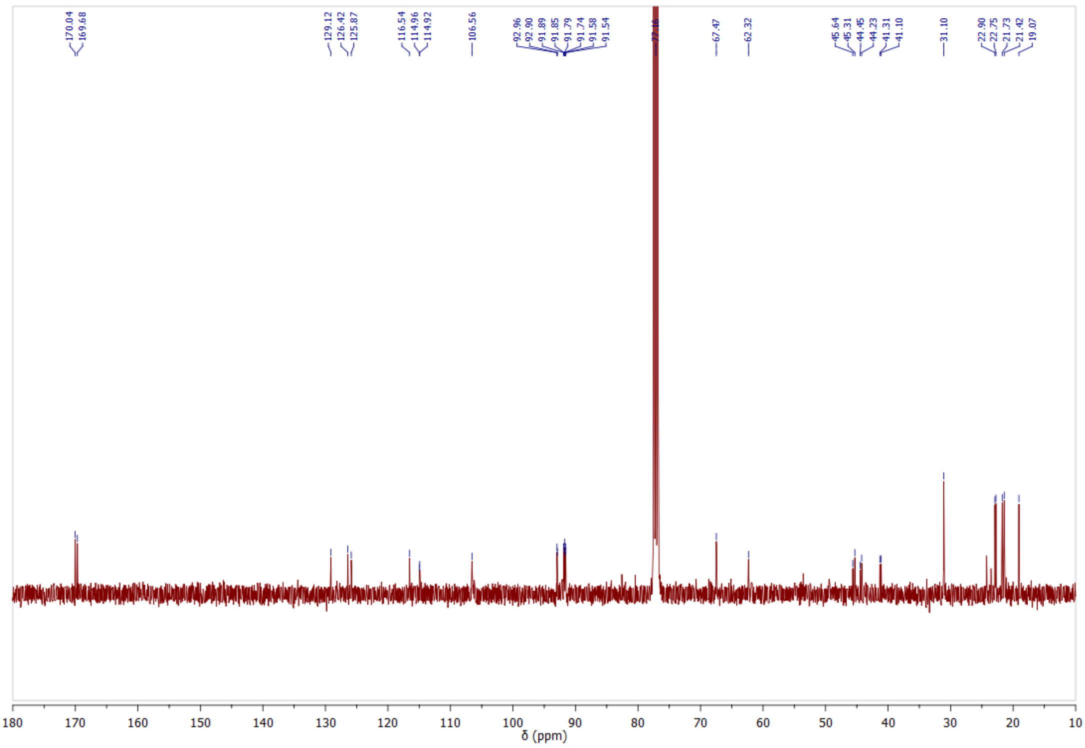

Figure S5. ^{13}C NMR spectrum (100 MHz, $CDCl_3$) of complex **5**.

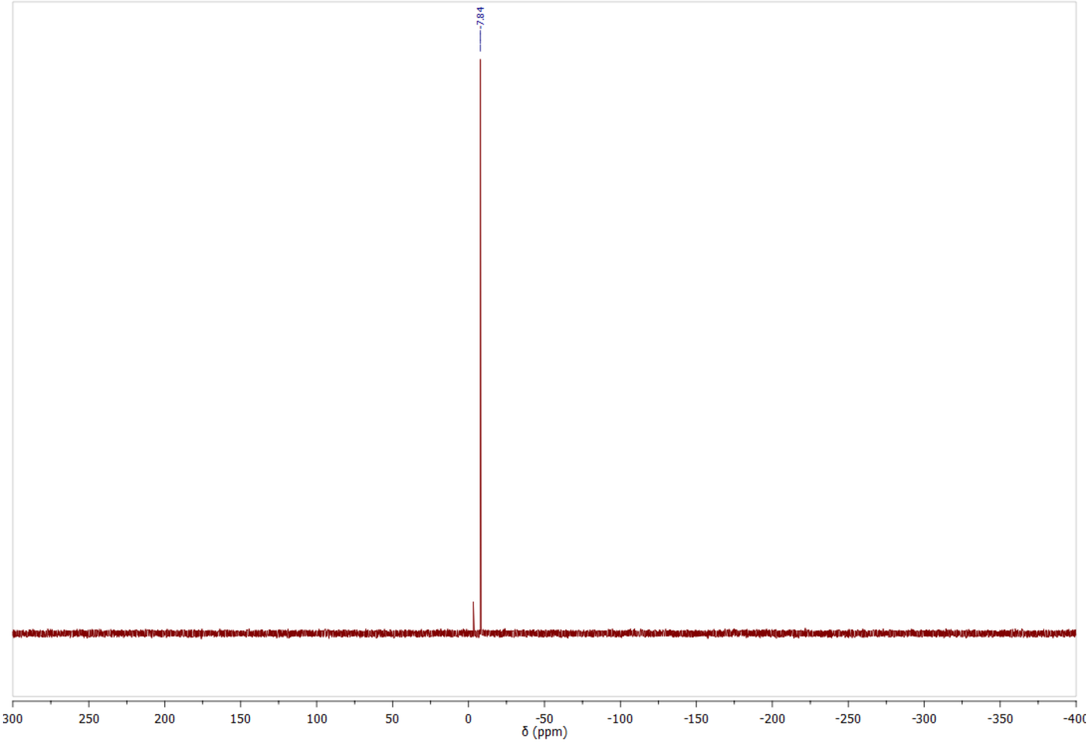

Figure S6. ^{31}P NMR spectrum (161 MHz, $CDCl_3$) of complex **5**.

c. NMR spectra for complex **6**

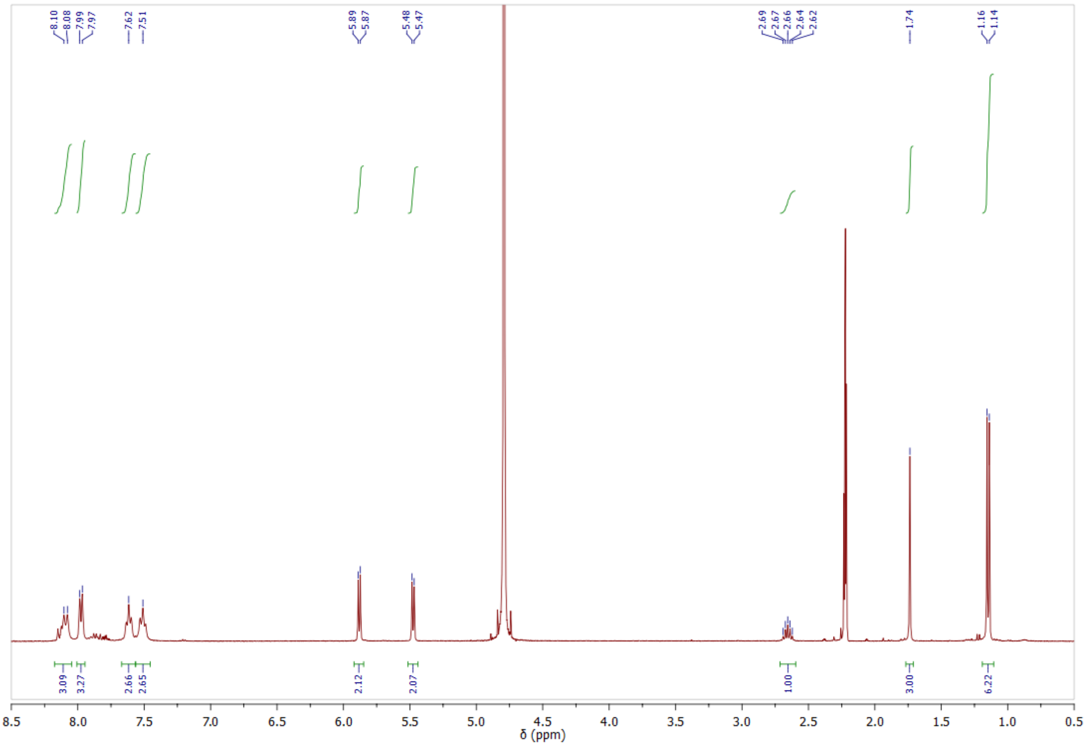

Figure S7. ^{1}H NMR spectrum (400 MHz, D_2O) of complex **6**.

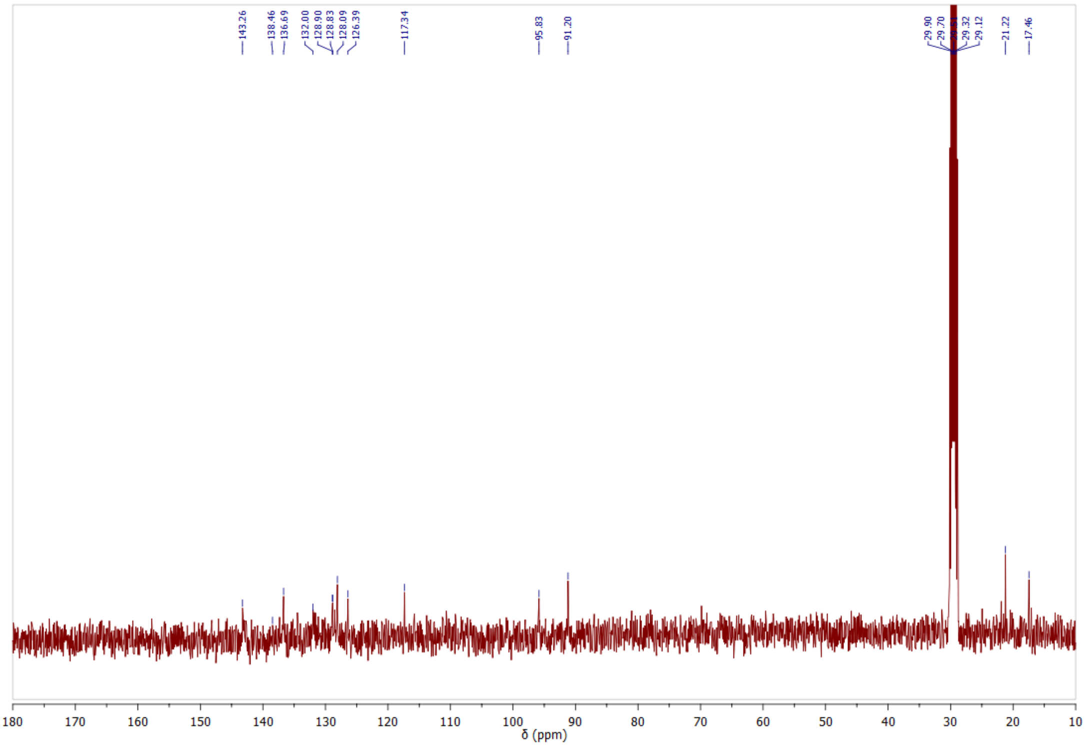

Figure S8. ^{13}C NMR spectrum (100 MHz, D_2O) of complex **6**.

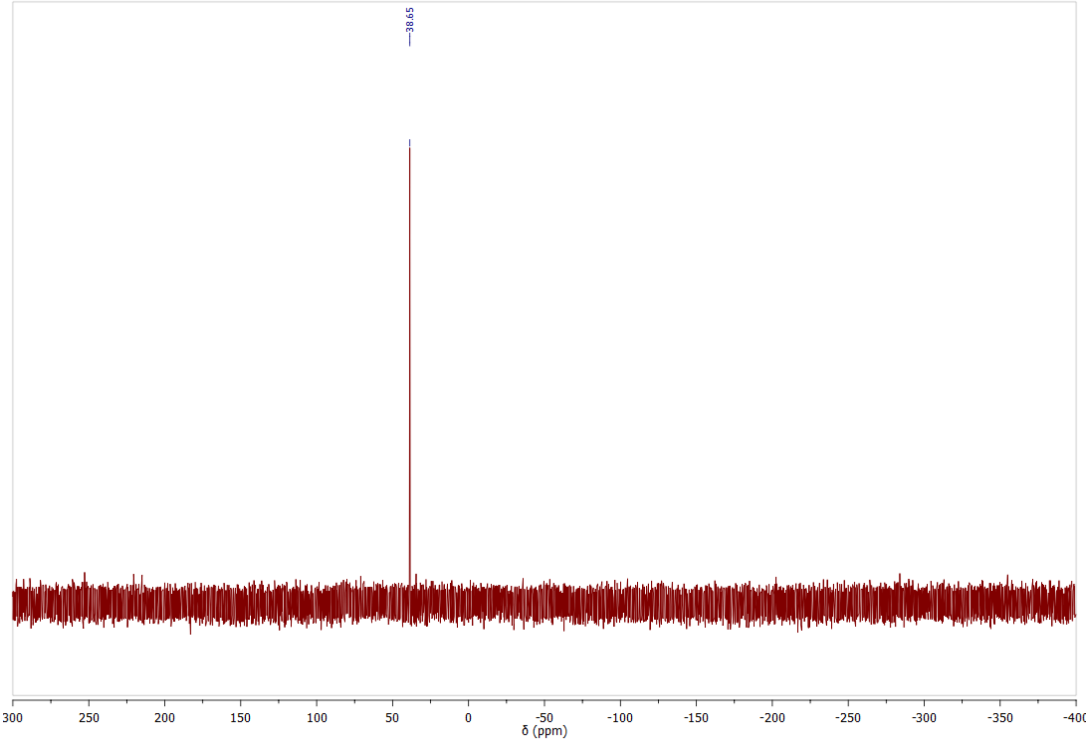

Figure S9. ^{31}P NMR spectrum (161 MHz, D_2O) of complex **6**.

d. NMR spectra for complex **7**

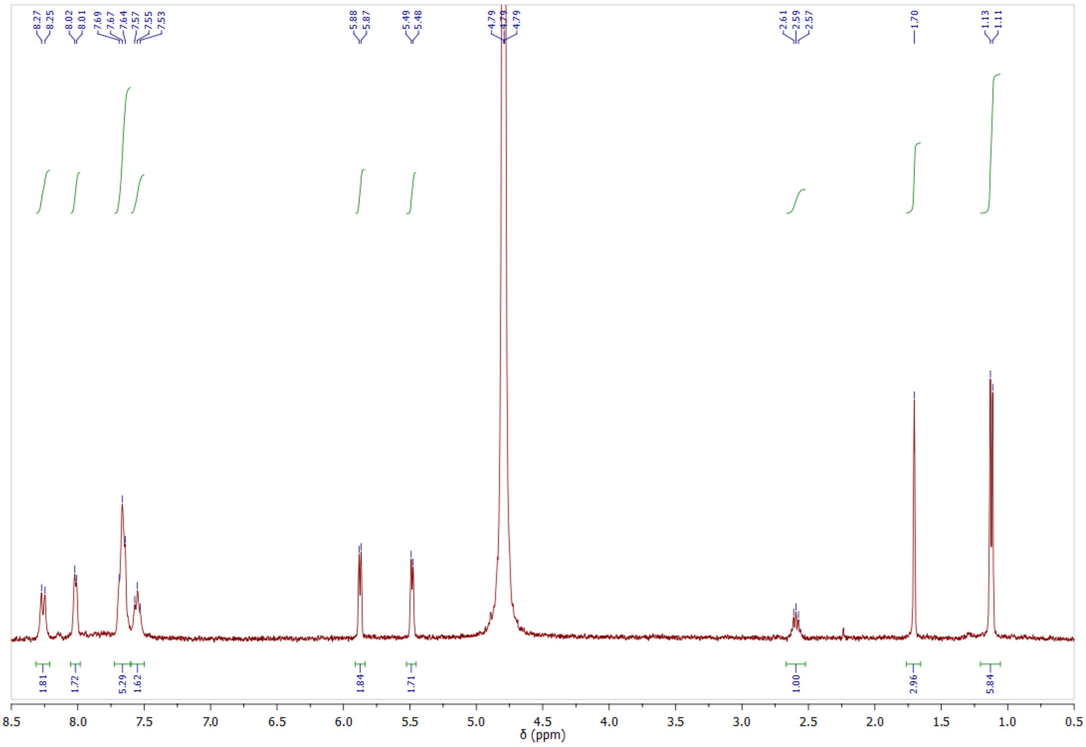

Figure S10. ^{1}H NMR spectrum (400 MHz, D_2O) of complex **7**.

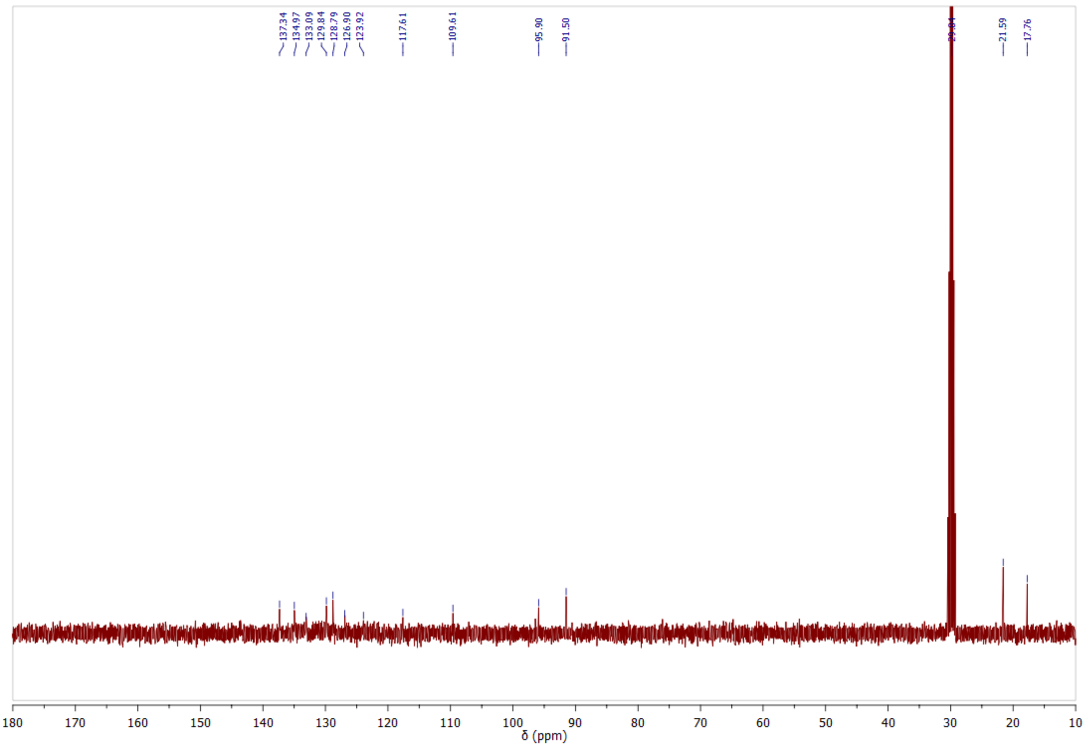

Figure S11. ^{13}C NMR spectrum (100 MHz, D_2O) of complex **7**.

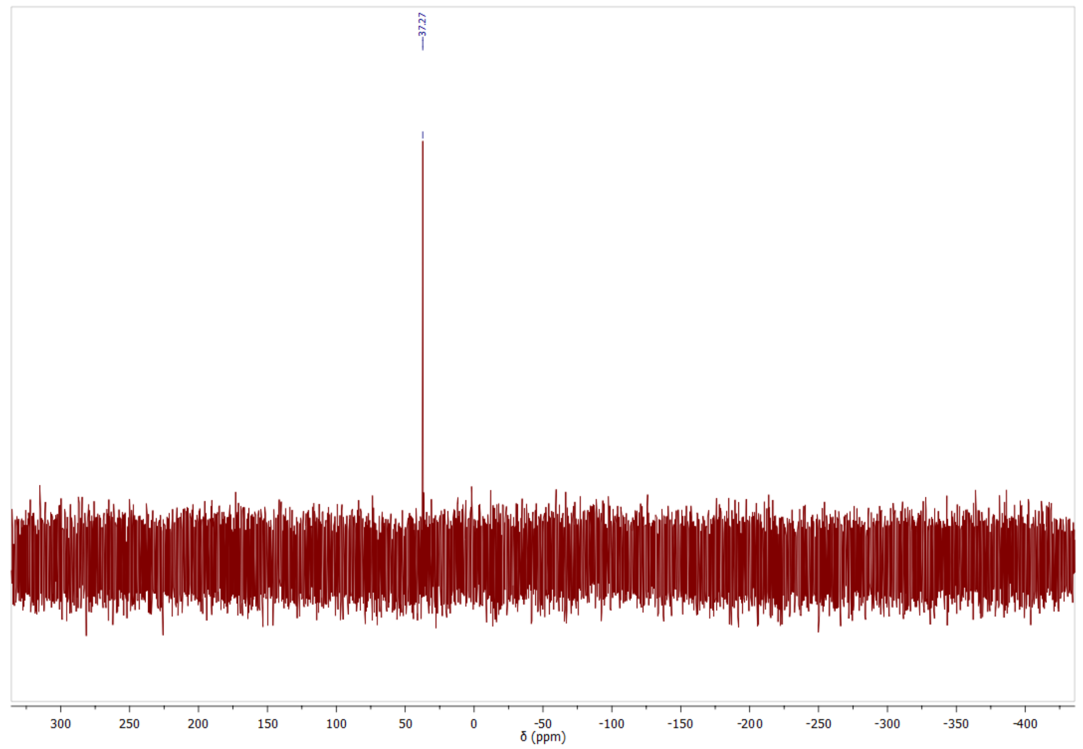

Figure S12. ^{31}P NMR spectrum (161 MHz, D_2O) of complex **7**.

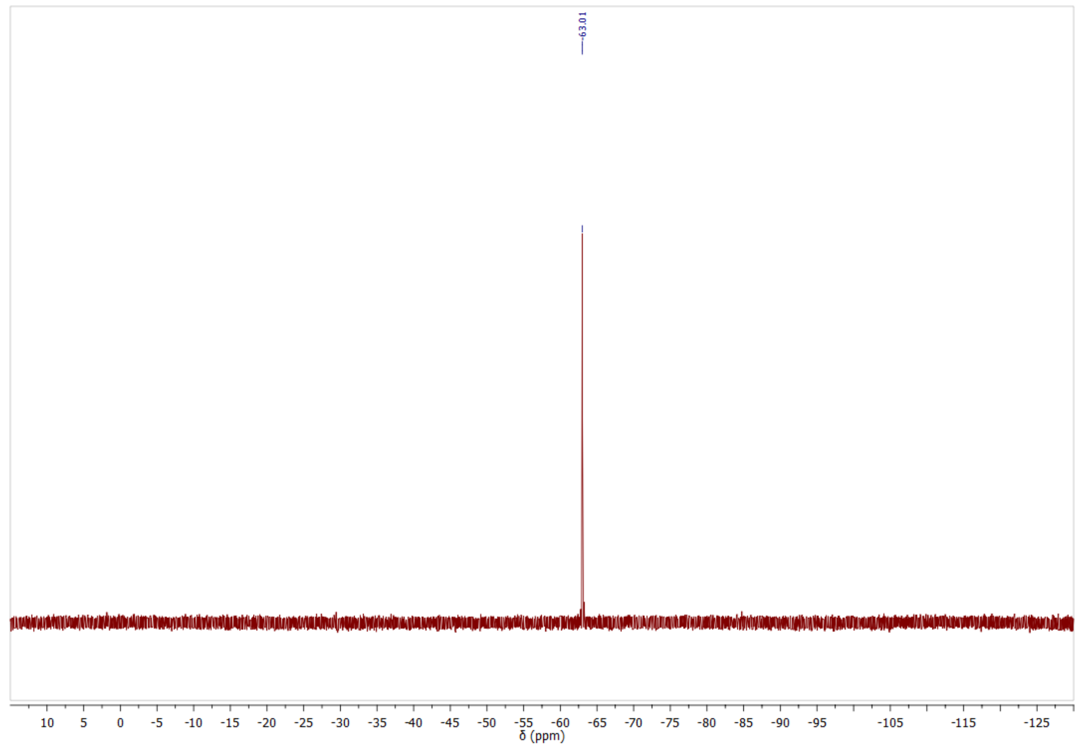

Figure S13. ^{19}F NMR spectrum (376 MHz, D_2O) of complex **7**.

e. NMR spectra for complex **8**

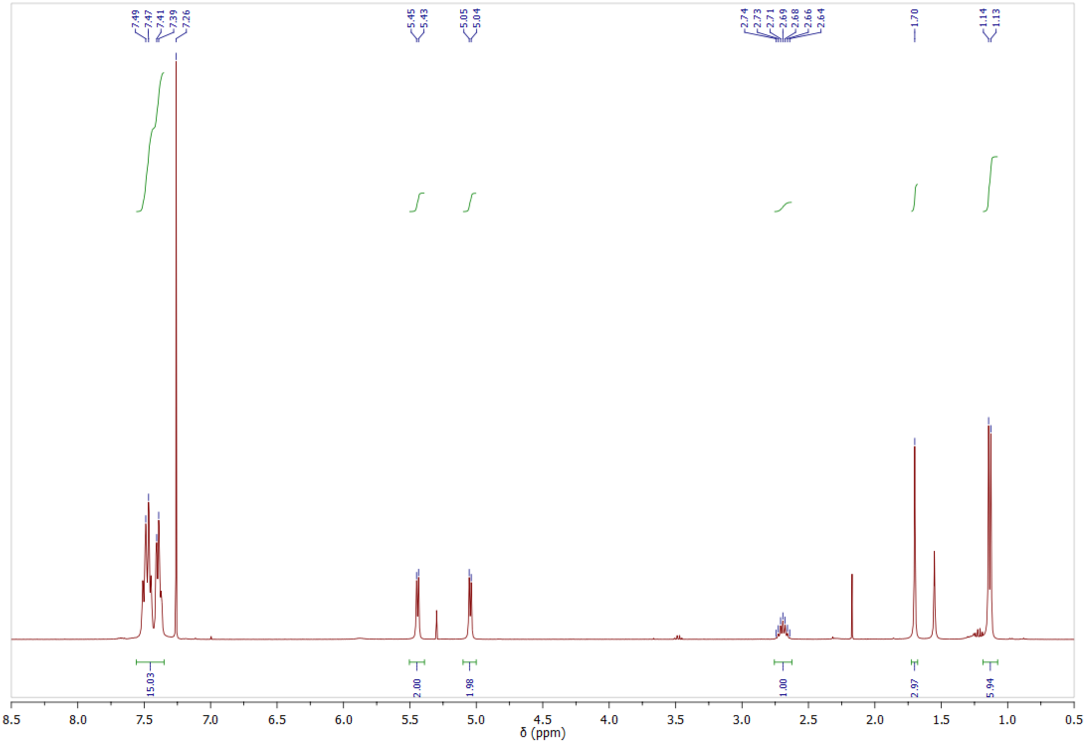

Figure S14. ^{1}H NMR spectrum (400 MHz, $CDCl_3$) of complex **8**.

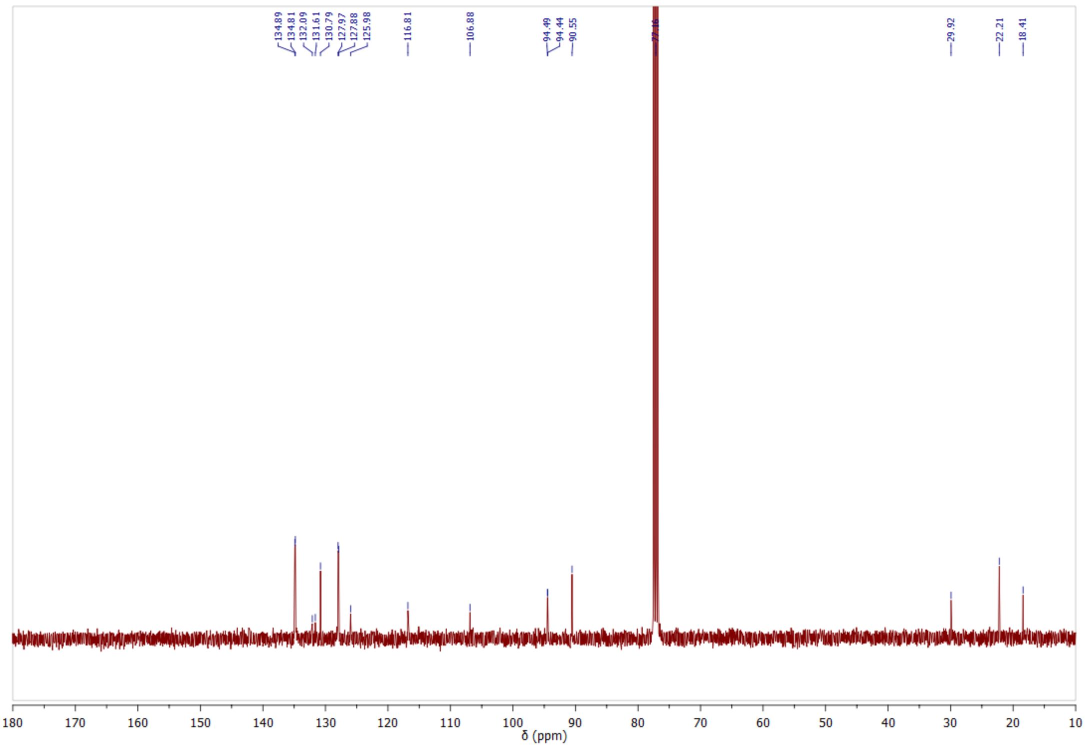

Figure S15. ^{13}C NMR spectrum (100 MHz, $CDCl_3$) of complex **8**.

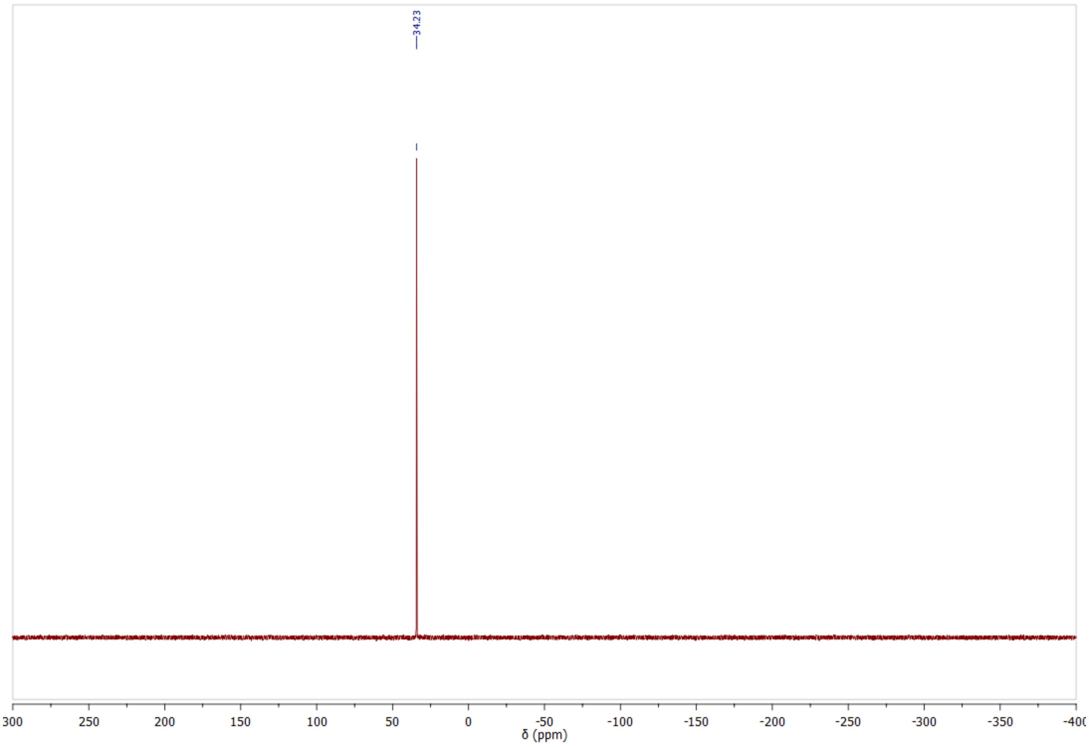

Figure S16. ^{31}P NMR spectrum (161 MHz, $CDCl_3$) of complex **8**.

II. IR spectra for complexes **4 – 8**

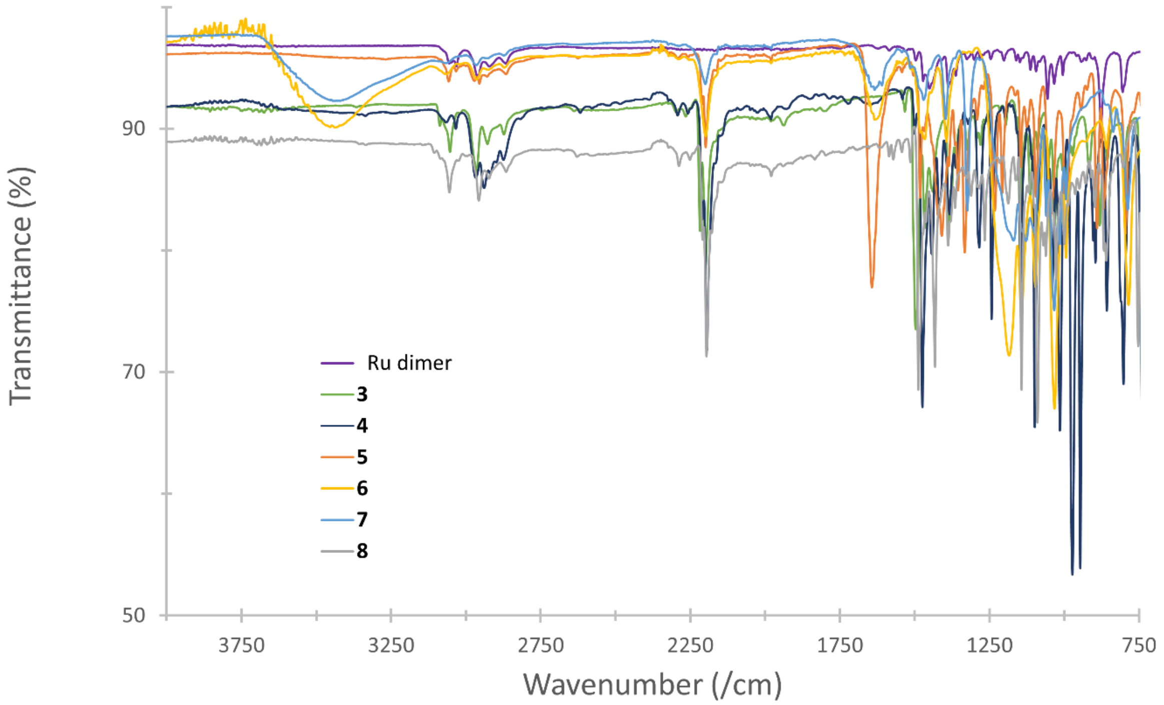

Figure S17. FT-IR spectra of complexes **3 – 8** and Ru dimer in the solid state.

III. HR-MS spectra for complexes **4 – 8**

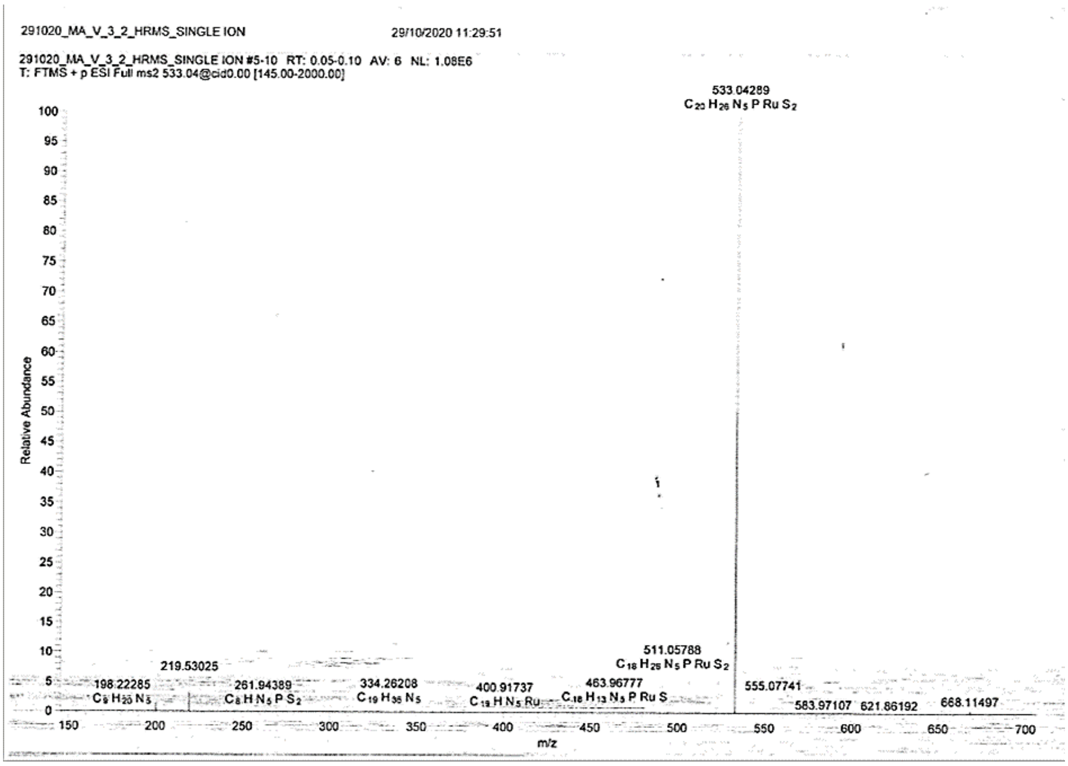

Figure S18. HR-ESI MS of complex **4**.

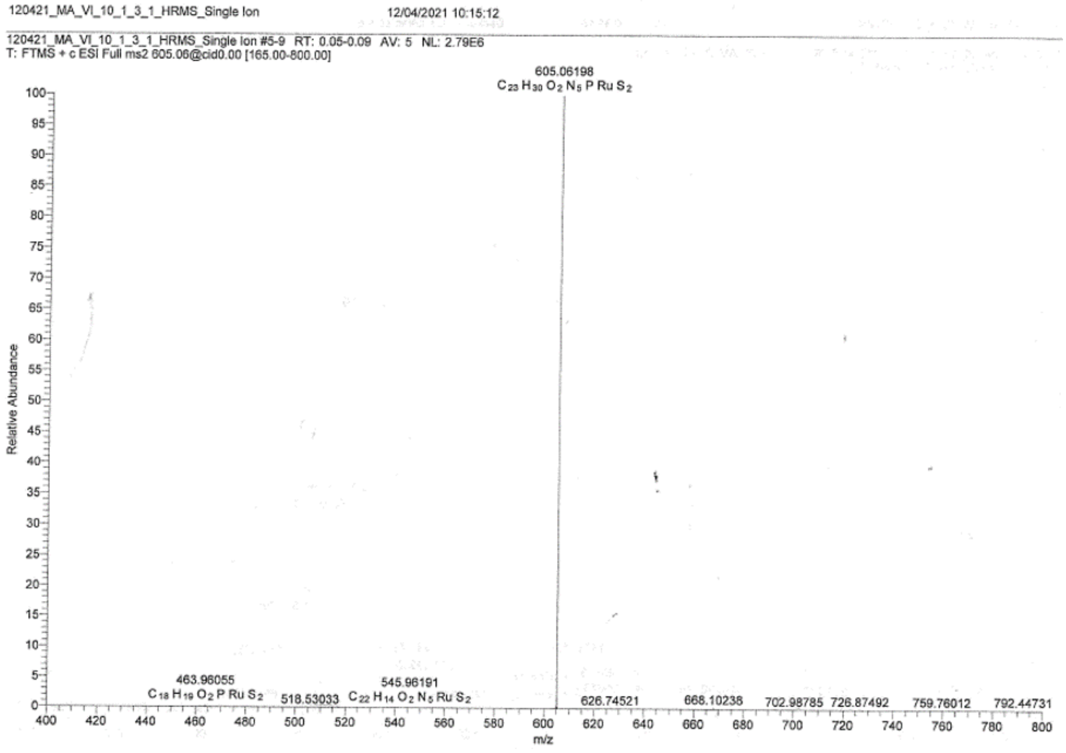

Figure S19. HR-ESI MS of complex **5**.

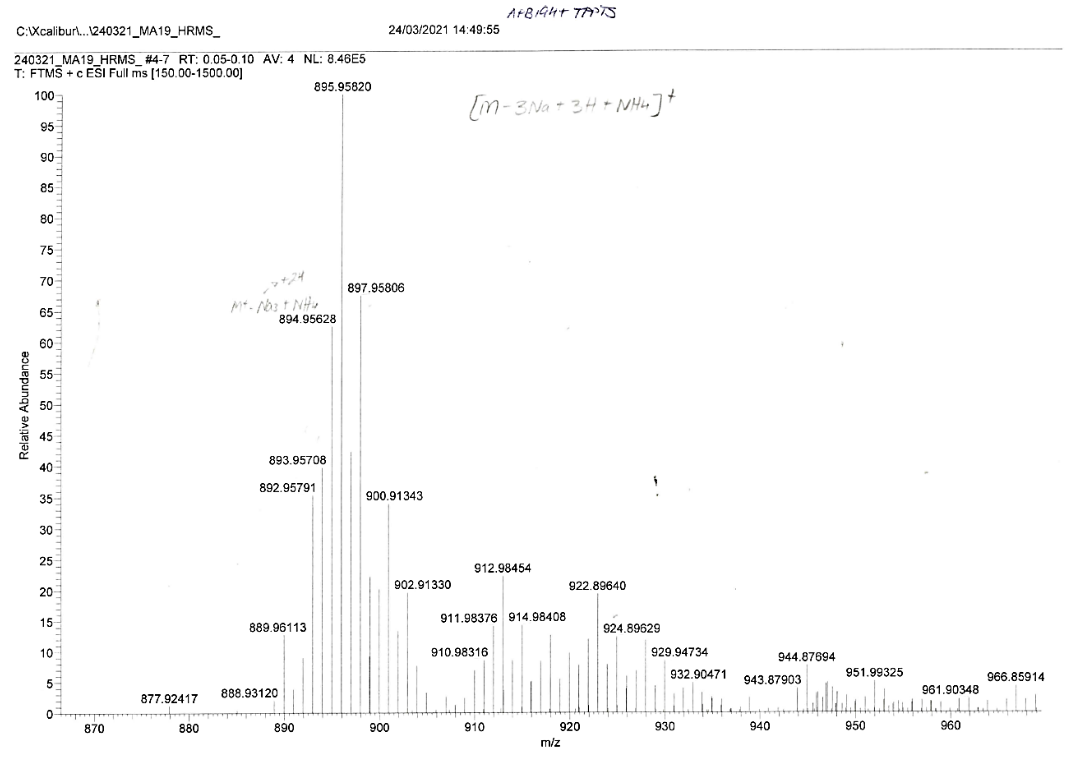

Figure S20. HR-ESI MS of complex **6**.

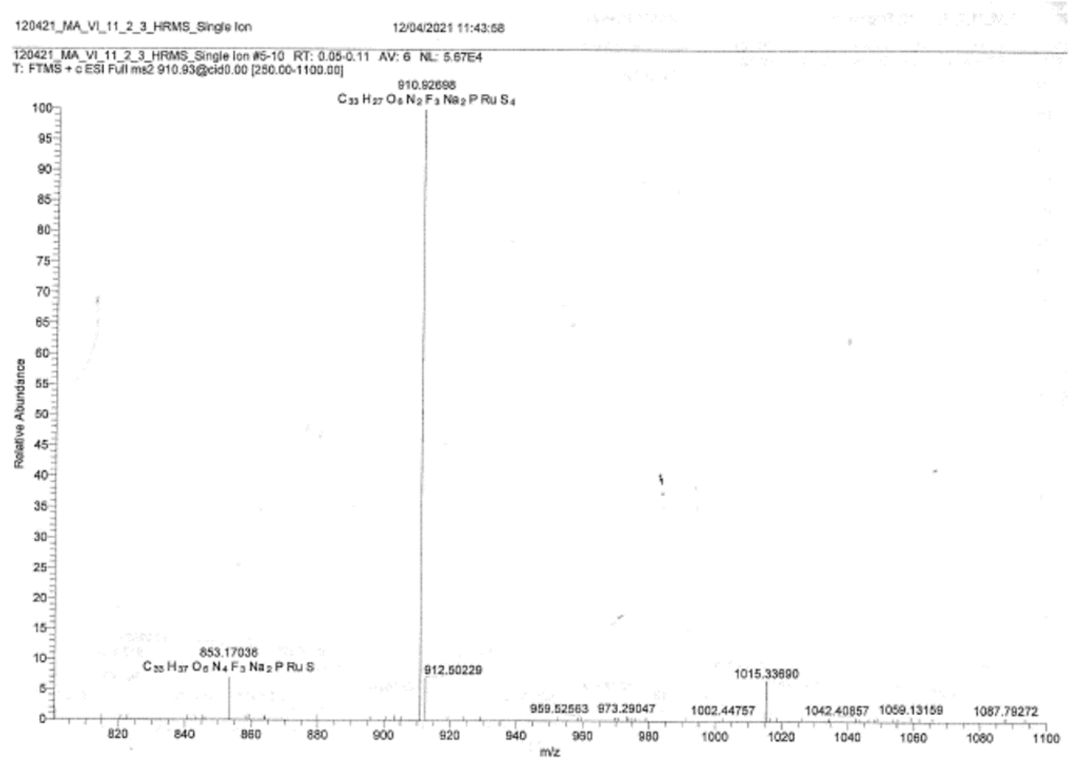

Figure S21. HR-ESI MS of complex **7**.

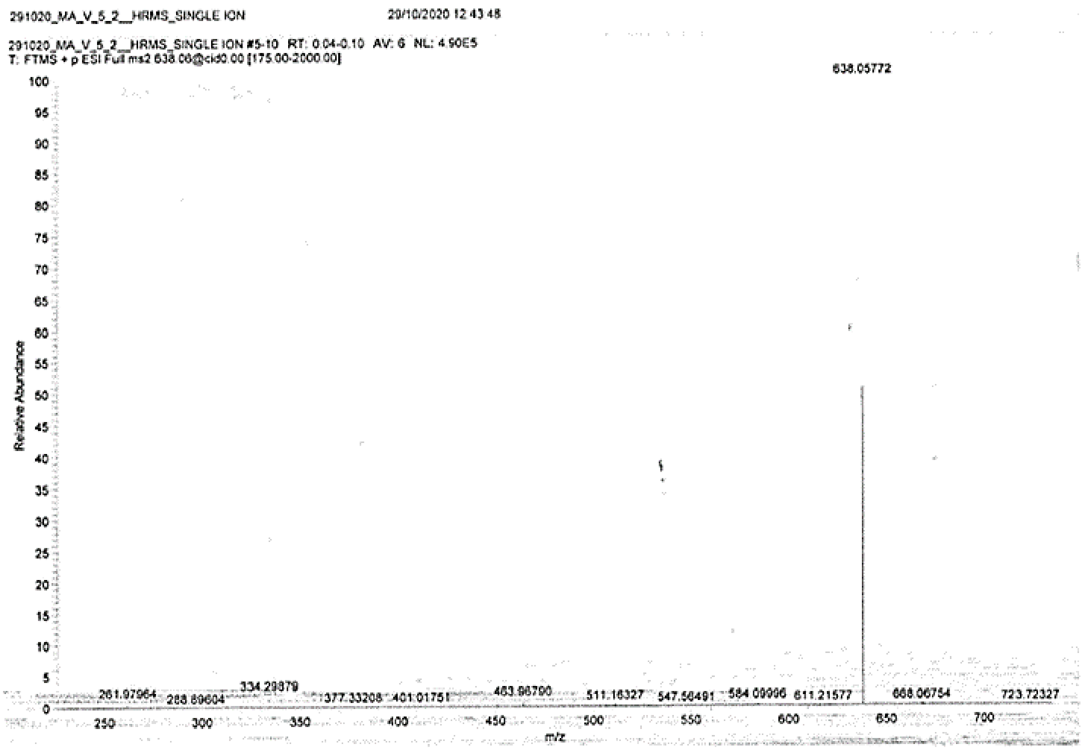

Figure S22. HR-ESI MS of complex **8**.

IV. XRD data for complex **4**

Deposition Number 2169506 contains the supplementary crystallographic data for this paper. These data are provided free of charge by the joint Cambridge Crystallographic Data Centre and Fachinformationszentrum Karlsruhe Access Structures service.

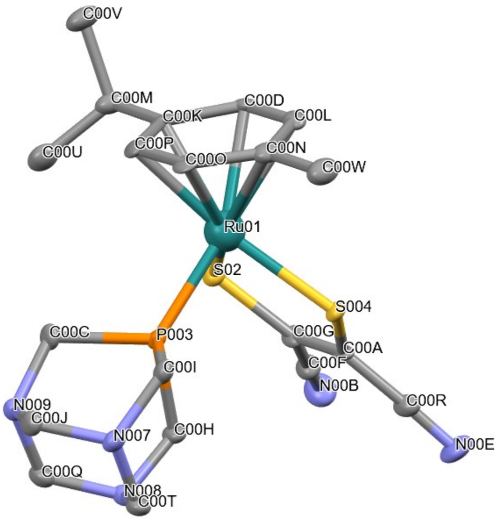

Figure S23. Molecular structure of complex **4**; all hydrogen atoms and the solvent molecule are omitted for clarity.

Table S1. Crystal data and structure refinement for complex **4**

Identification code	MA_VI_3_2_0m
Empirical formula	$C_{21}H_{28}Cl_2N_5PRuS_2$
Formula weight	617.54
Temperature/K	100.11
Crystal system	triclinic
Space group	P-1
a/Å	9.9054(14)
b/Å	10.0322(15)
c/Å	14.333(2)
α/°	69.575(8)
β/°	72.521(9)
γ/°	81.556(9)
Volume/Å^3	1271.7(3)
Z	2
ρ_{calc}g/cm^3	1.613
μ/mm^{-1}	1.074
F(000)	628.0

Crystal size/mm^3	0.41 × 0.06 × 0.04
Radiation	MoKα (λ = 0.71073)
2Θ range for data collection/°	5.428 to 52.704
Index ranges	-10 ≤ h ≤ 12, -12 ≤ k ≤ 12, -17 ≤ l ≤ 17
Reflections collected	18338
Independent reflections	4855 [R_{int} = 0.0818, R_{sigma} = 0.1098]
Data/restraints/parameters	4855/0/292
Goodness-of-fit on F^2	0.995
Final R indexes [I>=2σ (I)]	R_1 = 0.0495, wR_2 = 0.0951
Final R indexes [all data]	R_1 = 0.0945, wR_2 = 0.1086
Largest diff. peak/hole / e $Å^{-3}$	0.76/-1.44

Table S2. Selected bond lengths (Å) for complex **4**

Atoms	Length (Å)
Ru01-S02	2.369(2)
Ru01-P003	2.279(1)
Ru01-S004	2.370(1)
Ru01-C00D	2.284(5)
Ru01-C00K	2.244(6)
Ru01-C00L	2.292(6)
Ru01-C00N	2.242(8)
Ru01-C00O	2.222(7)
Ru01-C00P	2.216(6)
S02-C00G	1.740(6)
S004-C00A	1.753(7)
C00A-C00G	1.36(1)

Table S3. Selected bond angles (°) for complex **4**

Atoms	Angle (°)
S02-Ru01-P003	87.46(6)
S02-Ru01-S004	87.98(6)
S02-Ru01-C00D	92.9(2)
S02-Ru01-C00K	90.1(2)
S02-Ru01-C00L	118.6(2)
S02-Ru01-C00N	154.8(2)
S02-Ru01-C00O	153.8(2)
S02-Ru01-C00P	116.3(2)

P003-Ru01-S004	85.13(6)
P003-Ru01-C00D	157.8(2)
P003-Ru01-C00K	120.6(2)
P003-Ru01-C00L	153.8(2)
P003-Ru01-C00N	117.6(2)
P003-Ru01-C00O	93.3(2)
P003-Ru01-C00P	94.7(2)
S004-Ru01-C00D	117.1(2)
S004-Ru01-C00K	154.1(2)
S004-Ru01-C00L	92.6(2)
S004-Ru01-C00N	91.3(2)
S004-Ru01-C00O	118.2(2)
S004-Ru01-C00P	155.7(2)
Ru01-S02-C00G	102.4(2)
Ru01-S004-C00A	102.3(2)
S004-C00A-C00G	123.3(5)
S02-C00G-C00A	123.9(5)

V. Stability in solution for complexes **4 – 8**

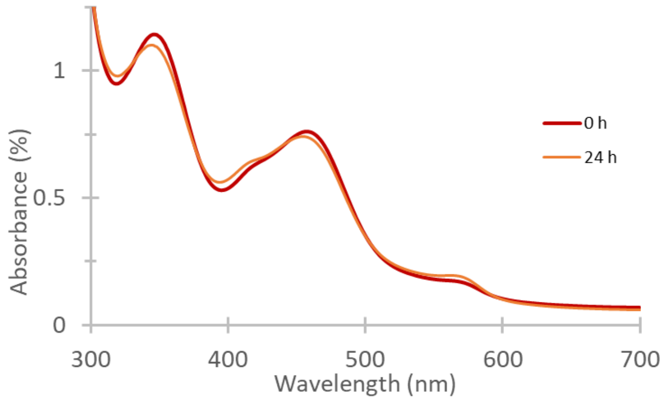

Figure S24. UV-Vis spectrum of complex **4** in a mixture DMSO/RPMI 1:1 (v/v) over time (1.5 × 10^{-4} M, 298 K, 24 h).

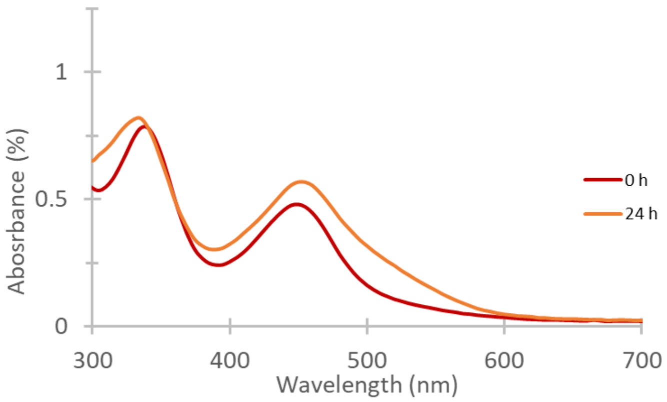

Figure S25. UV-Vis spectrum of complex **5** in a mixture DMSO/RPMI 1:99 (v/v) over time (10^{-4} M, 298 K, 24 h).

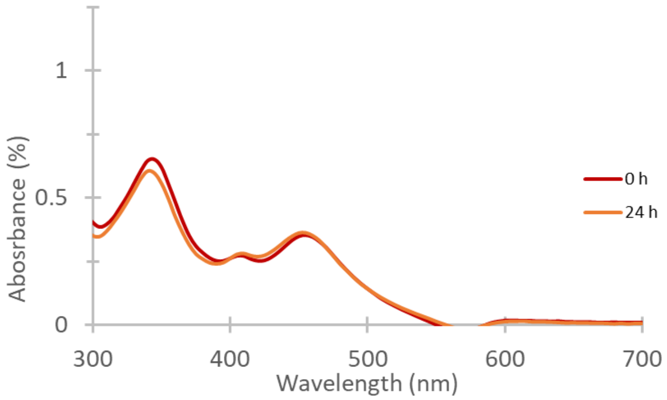

Figure S26. UV-Vis spectrum of complex **6** in RPMI over time (7.5×10^{-5} M, 298 K, 24 h).

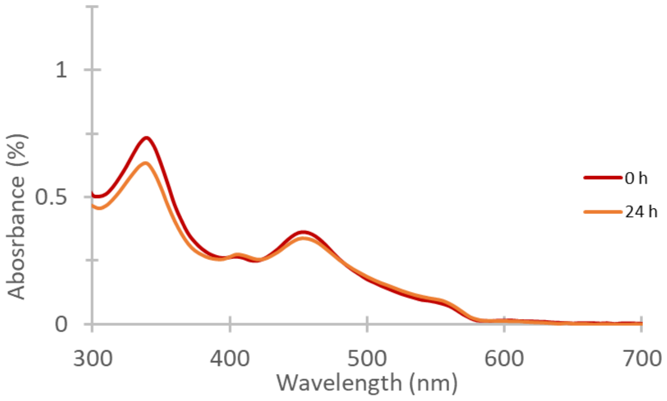

Figure S27. UV-Vis spectrum of complex **7** in RPMI over time (8×10^{-5} M, 298 K, 24 h).

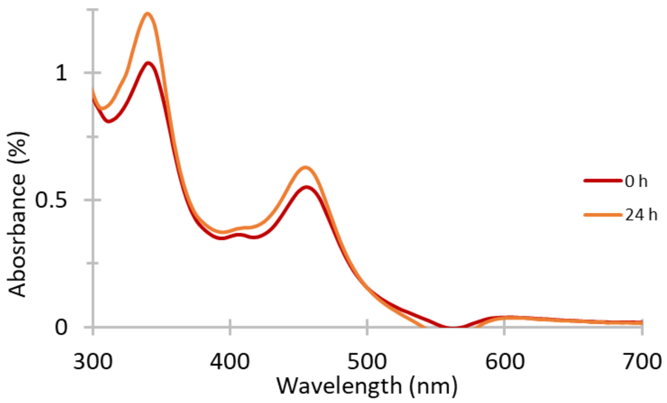

Figure S28. UV-Vis spectrum of complex **8** in a mixture DMSO/RPMI 1:99 (v/v) over time (10^{-4} M, 298 K, 24 h).

VI. *In vitro* antiproliferative activity for complexes **4 – 8**

a. IC_{50} data

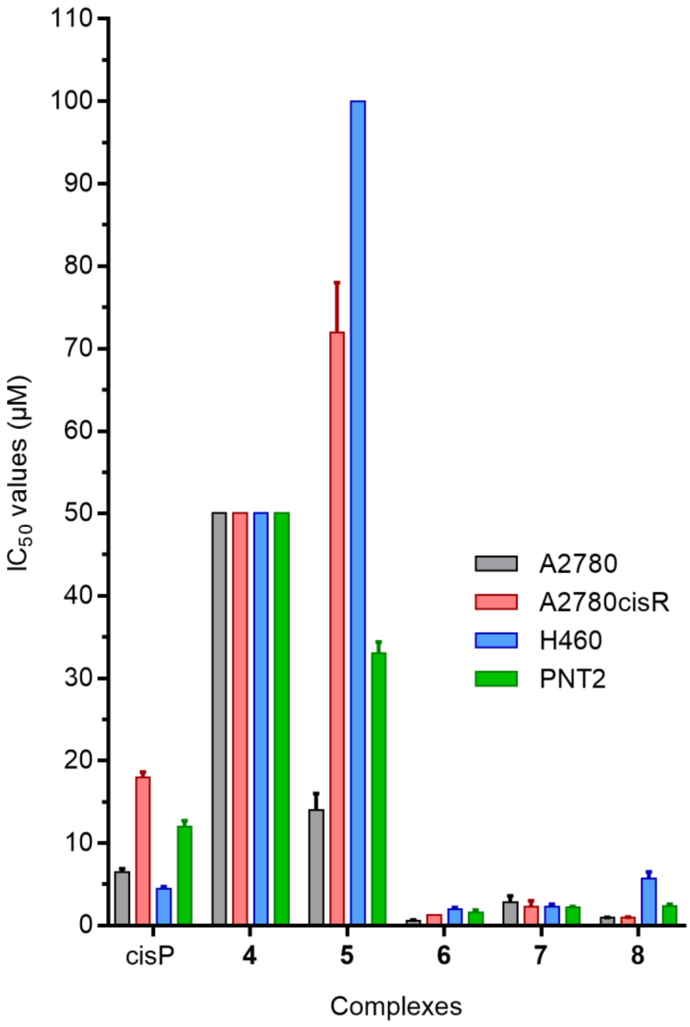

Figure S29. Bar-charts showing the IC_{50} values of cisplatin and complexes **4 – 8** against A2780, A2780cisR, H460, and PNT2.

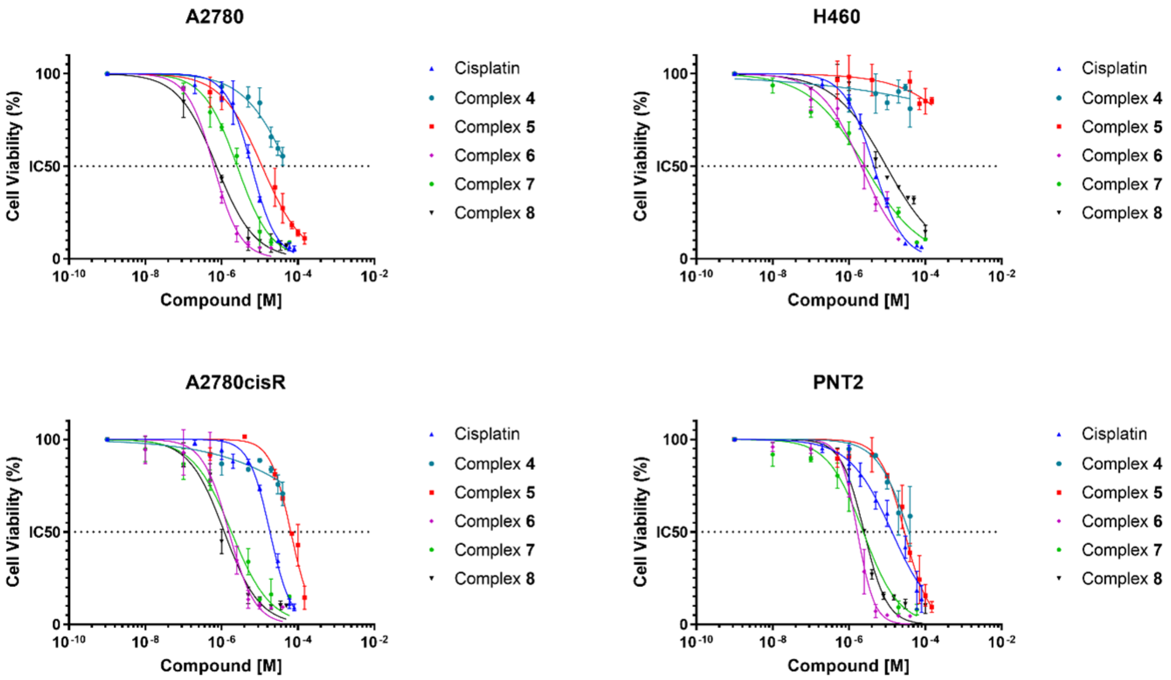

Figure S30. Dose-response curves used to generate IC_{50} values for complexes **4 – 8** against A2780, A2780cisR, H460, and PNT2.

b. IC_{50} data of ligands

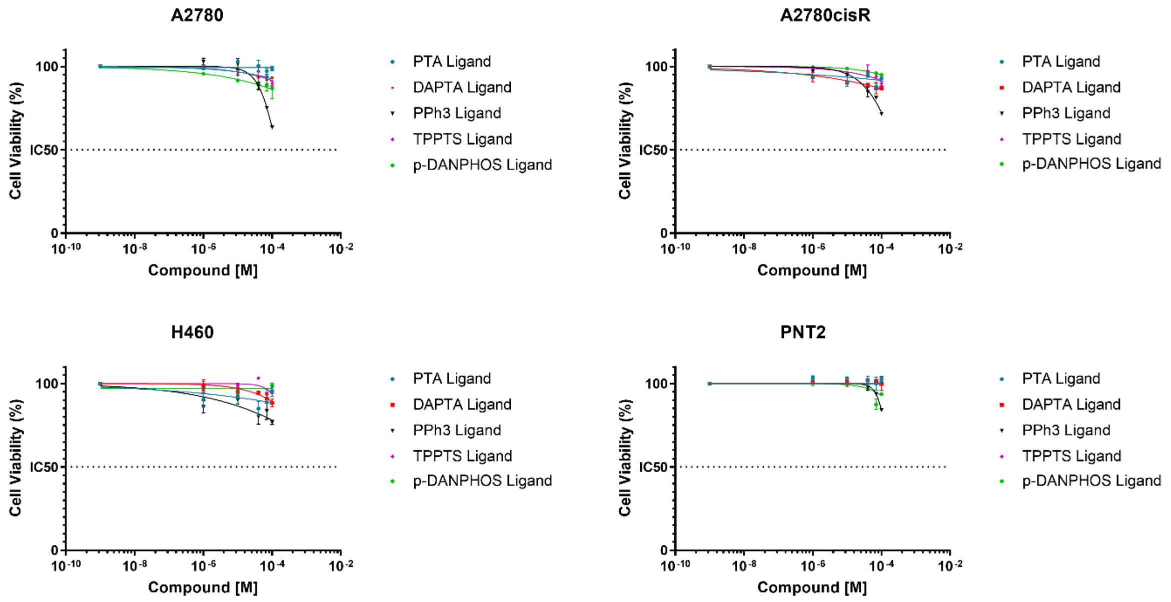

Figure S31. Dose-response curves used to generate IC_{50} values for phosphine ligands against A2780, A2780cisR, H460, and PNT2.

Table S4. IC_{50} values (µM) of phosphine ligands against cancerous ovarian cancer (A2780), cisplatin-resistant ovarian cancer (A2780cisR), non-small-cell lung cancer (H460) and non-cancerous human prostate (PNT2) cells.

Ligands	IC50 values (µM) ± SD			
	A2780	A2780cisR	H460	PNT2
PTA	≥ 100	≥ 100	≥ 100	≥ 100
DAPTA	≥ 100	≥ 100	≥ 100	≥ 100
PTA	≥ 100	≥ 100	≥ 100	≥ 100
TPPTS	≥ 100	≥ 100	≥ 100	≥ 100
p-DANPHOS	≥ 100	≥ 100	≥ 100	≥ 100

c. Selectivity index (SI)

Table S5. Selectivity indices for cisplatin and complexes **4 – 8** against cancerous ovarian cancer (A2780), cisplatin-resistant ovarian cancer (A2780cisR), and non-small-cell lung cancer (H460) cells.

Compounds	A2780	A2780cisR	H460
Cisplatin	1.85	0.67	2.67
**4**[a]	-	-	-
**5**	2.36	0.49	-
**6**	2.67	1.23	0.80
**7**	0.79	0.96	0.96
**8**	2.50	2.45	0.42

[a]Complex **4** precipitates at concentrations higher than 50 µM and IC_{50} values cannot be determined.

Table S6. Cross-resistance profiles of cisplatin and complexes **4 – 8** in cisplatin resistant A2780cisR cells compared to parental A2780 cells.

Compounds	Selectivity factor
Cisplatin	2.8
**4**[a]	-
**5**	5.1
**6**	2.2
**7**	0.82
**8**	1.02

[a]Complex **4** is precipitating at concentrations higher than 50 µM and IC_{50} values cannot be determined.

d. ROS

Table S7. ROS results

**Untreated**		Experiment 1	Experiment 2	Experiment 3	**Average**	Error
	Q1-LL	97.3	97.4	97.5	**97.4**	0.1
	Q1-LR	0.5	0.5	0.4	**0.5**	0.1
	Q1-UR	2.1	1.8	1.9	**1.9**	0.2
	Q1-UL	0.1	0.3	0.2	**0.2**	0.1
**H2O2**		Experiment 1	Experiment 2	Experiment 3	**Average**	Error
	Q1-LL	4.8	6.4	6.7	**6.0**	1.0
	Q1-LR	8.0	7.4	4.9	**6.8**	1.6
	Q1-UR	86.7	85.8	87.8	**86.8**	1.0
	Q1-UL	0.4	0.5	0.6	**0.5**	0.1
**Complex 6**		Experiment 1	Experiment 2	Experiment 3	**Average**	Error
	Q1-LL	4.0	4.2	4.5	**4.2**	0.3
	Q1-LR	19.0	17.0	15.1	**17.0**	2.0
	Q1-UR	77.0	78.7	80.3	**78.7**	1.7
	Q1-UL	0.0	0.1	0.1	**0.1**	0.1
**Complex 8**		Experiment 1	Experiment 2	Experiment 3	**Average**	Error
	Q1-LL	3.6	3.2	3.3	**3.4**	0.2
	Q1-LR	30.7	27.5	27.9	**28.7**	1.7
	Q1-UR	65.7	69.3	68.7	**67.9**	1.9
	Q1-UL	0.0	0.0	0.0	**0.0**	0.0

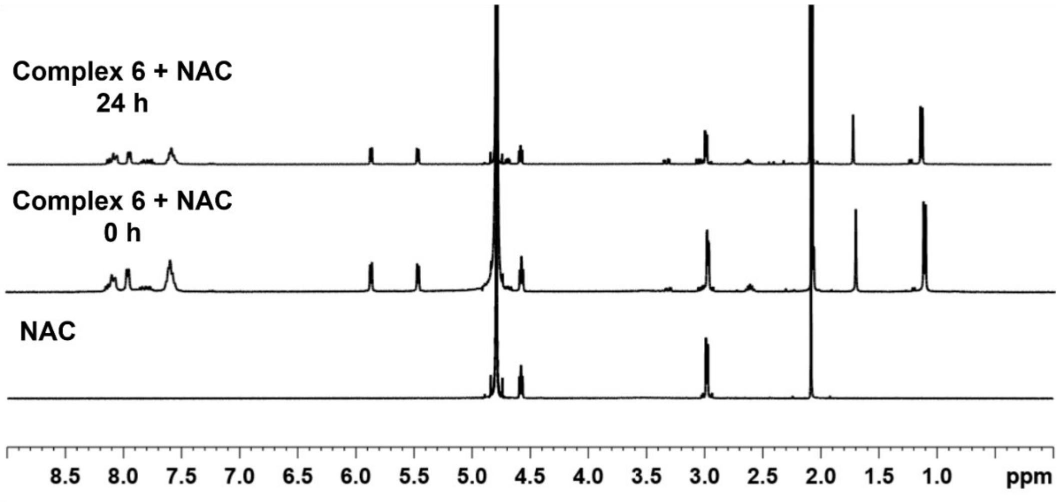

Figure S32. ^{1}H NMR spectra of complex **6** in the presence of 2 mol. equiv. of NAC over a period of 0 – 24 h (1.1 mM complex,D_2O, 298 K).

e. Mitochondrial-membrane potential

Table S8. Mitochondrial-membrane potential – Experiment 1

Sample	% cell population with high orange fluorescence	% cell population with low orange fluorescence
Untreated	93.7	6.3
cisplatin (6.5 μM)	88.9	11.1
Complex **6** (IC_{50})	60.4	39.6
Complex **8** (IC_{50})	81.9	18.1

Table S9. Mitochondrial-membrane potential – Experiment 2

Sample	% cell population with high orange fluorescence	% cell population with low orange fluorescence
Untreated	93.2	6.8
cisplatin (6.5 μM)	87	13.0
Complex **6** (IC_{50})	55.5	44.5
Complex **8** (IC_{50})	82.5	17.5

Table S10. Mitochondrial-membrane potential – Experiment 3

Sample	% cell population with high orange fluorescence	% cell population with low orange fluorescence
Untreated	93.6	6.4
cisplatin (6.5 μM)	82.1	17.9
Complex **6** (IC_{50})	62.7	37.3
Complex **8** (IC_{50})	80.0	20.0

Table S11. Mitochondrial-membrane potential – Summary of results (mean values ± SD)

Sample	% cell population with high orange fluorescence	% cell population with low orange fluorescence
Untreated	93.5 ± 0.3	6.5 ± 0.3
cisplatin (6.5 μM)	86 ± 3.5	16 ± 2
Complex **6** (IC_{50})	59.5 ± 3.7	40.5 ± 3.7
Complex **8** (IC_{50})	81.5 ± 1.3	18.5 ± 1.3

f. Apoptosis

Experiments were performed as duplicate of triplicates.

Table S12. Apoptosis results - Experiment 1.

		Rep 1	Rep 2	Rep 3	Average	Error
**Untreated**		Rep 1	Rep 2	Rep 3	**Average**	Error
	Viable cells (Q1-LL)	95.0	94.2	94.3	**94.5**	0.4
	Early apoptosis (Q1-LR)	0.3	0.5	0.5	**0.4**	0.1
	Late apoptosis (Q1-UR)	3.5	3.6	3.8	**3.6**	0.2
	Necrosis (Q1-UL)	1.2	1.7	1.5	**1.5**	0.3
**Doxorubicin (0.5 µM)**		Rep 1	Rep 2	Rep 3	**Average**	Error
	Viable cells (Q1-LL)	0.0	0.0	0.0	**0.0**	0.0
	Early apoptosis (Q1-LR)	0.0	0.0	0.0	**0.0**	0.0
	Late apoptosis (Q1-UR)	99.8	99.9	100.0	**99.9**	0.1
	Necrosis (Q1-UL)	0.2	0.1	0.0	**0.1**	0.1
**Complex 6 (2xIC50)**		Rep 1	Rep 2	Rep 3	**Average**	Error
	Viable cells (Q1-LL)	0.2	0.2	0.3	**0.2**	0.1
	Early apoptosis (Q1-LR)	0.1	0.1	0.2	**0.1**	0.1
	Late apoptosis (Q1-UR)	99.6	99.6	99.5	**99.6**	0.1
	Necrosis (Q1-UL)	0.0	0.0	0.0	**0.0**	0.0
**Complex 8 (2xIC50)**		Rep 1	Rep 2	Rep 3	**Average**	Error
	Viable cells (Q1-LL)	1.7	2.8	3.5	**2.7**	0.9
	Early apoptosis (Q1-LR)	2.6	2.2	3.0	**2.6**	0.4
	Late apoptosis (Q1-UR)	95.4	94.5	93.0	**94.3**	1.2
	Necrosis (Q1-UL)	0.3	0.4	0.6	**0.4**	0.2

Table S13. Apoptosis results - Experiment 2.

**Untreated**		Rep 1	Rep 2	Rep 3	**Average**	Error
	Viable cells (Q1-LL)	95.1	96.1	96.1	**95.8**	0.6
	Early apoptosis (Q1-LR)	0.6	0.5	0.3	**0.5**	0.2
	Late apoptosis (Q1-UR)	3.1	2.5	2.6	**2.7**	0.3
	Necrosis (Q1-UL)	1.2	0.9	1.0	**1.0**	0.2
**Doxorubicin (0.5 µM)**		Rep 1	Rep 2	Rep 3	**Average**	Error
	Viable cells (Q1-LL)	0.0	0.1	0.0	**0.0**	0.1
	Early apoptosis (Q1-LR)	0.0	0.0	0.0	**0.0**	0.0
	Late apoptosis (Q1-UR)	99.9	99.9	100.0	**99.9**	0.1
	Necrosis (Q1-UL)	0.0	0.1	0.0	**0.0**	0.1
**Complex 6 (2xIC50)**		Rep 1	Rep 2	Rep 3	**Average**	Error
	Viable cells (Q1-LL)	0.1	0.0	0.1	**0.1**	0.1
	Early apoptosis (Q1-LR)	0.0	0.0	0.0	**0.0**	0.0
	Late apoptosis (Q1-UR)	99.9	99.9	99.9	**99.9**	0.0
	Necrosis (Q1-UL)	0.0	0.0	0.0	**0.0**	0.0
**Complex 8 (2xIC50)**		Rep 1	Rep 2	Rep 3	**Average**	Error
	Viable cells (Q1-LL)	2.3	2.7	2.5	**2.5**	0.2
	Early apoptosis (Q1-LR)	2.0	2.3	2.9	**2.4**	0.5
	Late apoptosis (Q1-UR)	95.1	94.2	94.1	**94.5**	0.6
	Necrosis (Q1-UL)	0.6	0.8	0.5	**0.6**	0.2

Table S14. Apoptosis results - Combined results.

Summary from two experiments					
**Untreated**		Exp 1	Exp 2	**Average**	Error
	Viable cells (Q1-LL)	94.5	95.8	**95.2**	0.9
	Early apoptosis (Q1-LR)	0.4	0.5	**0.5**	0.1
	Late apoptosis (Q1-UR)	3.6	2.7	**3.2**	0.6
	Necrosis (Q1-UL)	1.5	1.0	**1.3**	0.4
**Doxorubicin (0.5 µM)**		Exp 1	Exp 2	**Average**	Error
	Viable cells (Q1-LL)	0.0	0.0	**0.0**	0.0
	Early apoptosis (Q1-LR)	0.0	0.0	**0.0**	0.0
	Late apoptosis (Q1-UR)	99.9	99.9	**99.9**	0.0
	Necrosis (Q1-UL)	0.1	0.0	**0.1**	0.1
**Complex 6 (2xIC50)**		Exp 1	Exp 2	**Average**	Error
	Viable cells (Q1-LL)	0.2	0.1	**0.2**	0.1
	Early apoptosis (Q1-LR)	0.1	0.0	**0.1**	0.1
	Late apoptosis (Q1-UR)	99.6	99.9	**99.8**	0.2
	Necrosis (Q1-UL)	0.0	0.0	**0.0**	0.0
**Complex 8 (2xIC50)**		Exp 1	Exp 2	**Average**	Error
	Viable cells (Q1-LL)	2.7	2.5	**2.6**	0.1
	Early apoptosis (Q1-LR)	2.6	2.4	**2.5**	0.1
	Late apoptosis (Q1-UR)	94.3	94.5	**94.4**	0.1
	Necrosis (Q1-UL)	0.4	0.6	**0.5**	0.1

VII. *In vivo* results for complexes 6 and 8

a. Evaluation of the maximum tolerated dose (MTD)

Three runs were carried out for the determination of MTD for the two complexes **6** and **8** (Figures S33-S35).

Complex **6**

Initially complex **6** was administered at 10mg/kg/dose in 10% DMSO/arachis oil (Figure S33). Toxicity was not observed at this dose and so the dose was increased to 20mg/kg for the next run (Figure S34). Again no toxicity was observed and so for the final run, 50mg/kg/dose was administered for the whole schedule (Figure S35). No evidence of toxicity was seen and so this was determined to be the MTD for the efficacy studies.

Complex **8**

Initially complex **8** was administered at 10mg/kg/dose in 10% DMSO/arachis oil (Figure S33). As increasing the dose to 20 mg/kg/dose resulted in issues with the complex failing to form a stable emulsion when the oil was added, the dose was subsequently reduced to 15mg/kg, with DMSO concentration increased to 20% for further dosing in this run. One animal had to be culled on day 8 but this was not related to the dosing. A full run was then carried out at 15mg/kg in 20% DMSO/arachis oil (Figure S35) and no evidence of toxicity was seen and so this was determined to be the MTD for the efficacy studies.

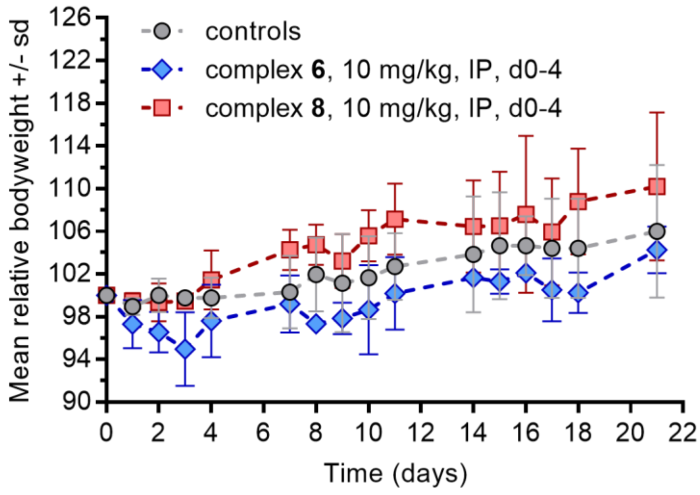

Figure S33. MTD evaluation for complexes **6** and **8** at 10 mg/kg/dose – run 1.

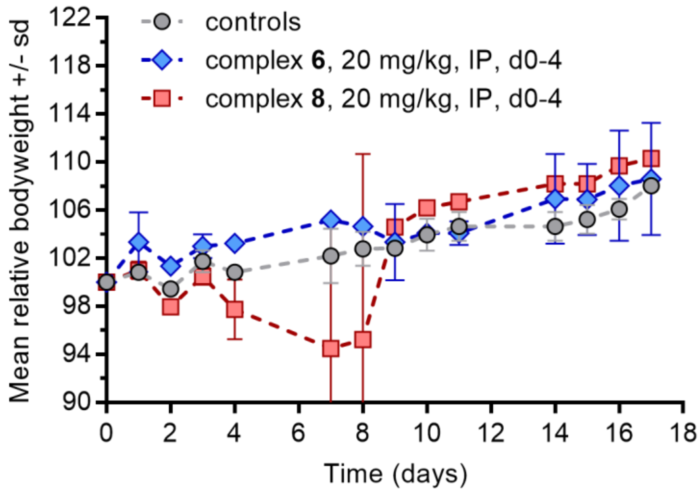

Figure S34. MTD evaluation for complexes **6** and **8** at 20 mg/kg/dose – run 2.

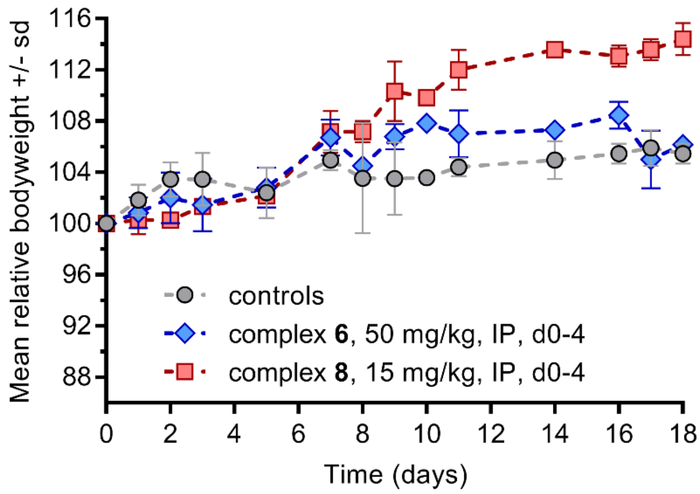

Figure S35. MTD evaluation for complexes **6** and **8** at 50 and 15 mg/kg/dose, respectively – run 3.

b. Efficacy with subcutaneous tumour xenograft models

Table S15. A2780 efficacy study results.

Group	Median time to RTV2 (days)	Growth delay (days)	Maximum % weight loss
**6**, 50 mg/kg, i.p.	2.0	0.6	0
**8**, 15 mg/kg, i.p.	1.5	0.1	0
untreated	1.4	-	0

Owing to small group sizes, it was not possible to determine the statistical significance.

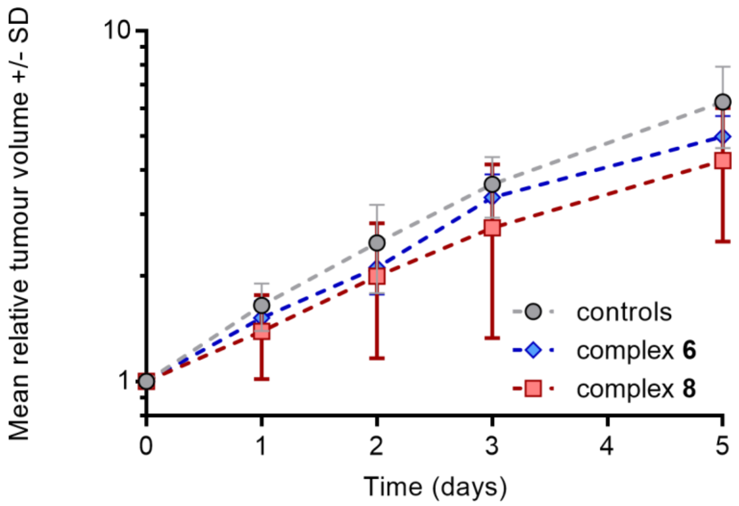

Figure S36. Xenograft study of the therapy of A2780 tumours with complexes **6** and **8** (mean relative tumour volume ± SD against time).

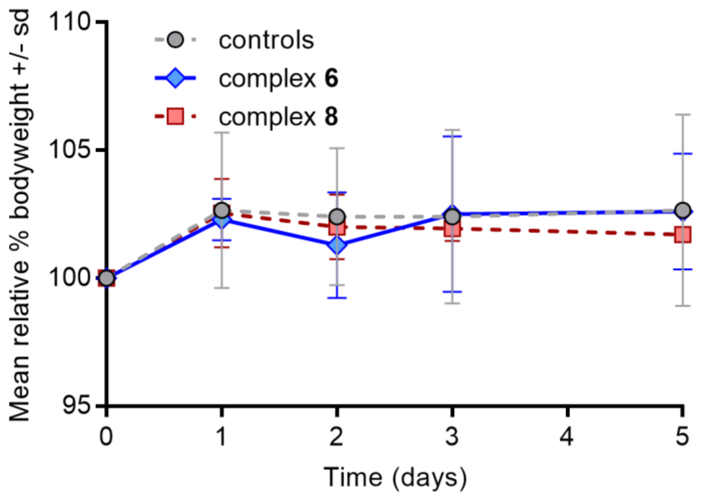

Figure S37. Relative bodyweight curves for A2780 tumour-bearing mice treated with complexes **6** and **8**.

Table S16. A2780/cis efficacy study results.

Group	Median time to RTV2 (days)	Growth delay (days)	Maximum % weight loss
**6**, 50 mg/kg, i.p.	3.0	0.2	2 (d3)
**8**, 15 mg/kg, i.p.	10.0	7.2	2 (d1)
untreated	2.8	-	1 (d1)

Owing to small group sizes, it was not possible to determine the statistical significance.

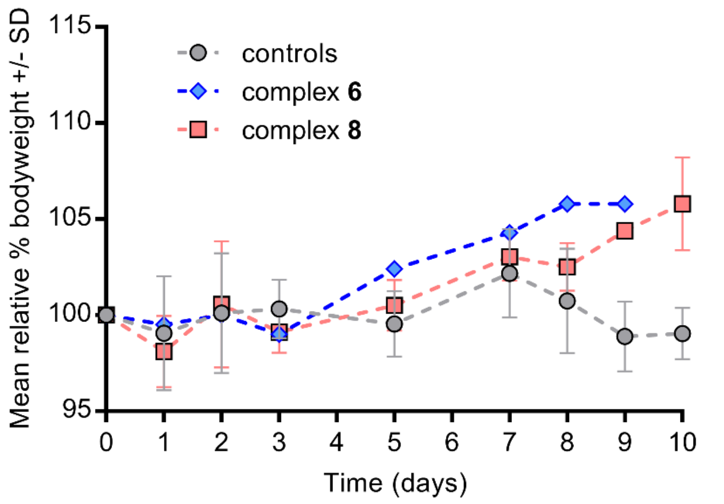

Figure S38. Relative bodyweight curves for A2780cisR tumour-bearing mice treated with complexes **6** and **8**.
